# Estimating the predictive ability of genetic risk models in simulated data based on published results from genome-wide association studies

**DOI:** 10.3389/fgene.2014.00179

**Published:** 2014-06-13

**Authors:** Suman Kundu, Raluca Mihaescu, Catherina M. C. Meijer, Rachel Bakker, A. Cecile J. W. Janssens

**Affiliations:** ^1^Department of Epidemiology, Erasmus University Medical CenterRotterdam, Netherlands; ^2^Department of Epidemiology, Rollins School of Public Health, Emory UniversityAtlanta, GA, USA

**Keywords:** predictive ability, risk prediction, modeling, genetic, AUC, GWAS

## Abstract

**Background:** There is increasing interest in investigating genetic risk models in empirical studies, but such studies are premature when the expected predictive ability of the risk model is low. We assessed how accurately the predictive ability of genetic risk models can be estimated in simulated data that are created based on the odds ratios (ORs) and frequencies of single-nucleotide polymorphisms (SNPs) obtained from genome-wide association studies (GWASs).

**Methods:** We aimed to replicate published prediction studies that reported the area under the receiver operating characteristic curve (AUC) as a measure of predictive ability. We searched GWAS articles for all SNPs included in these models and extracted ORs and risk allele frequencies to construct genotypes and disease status for a hypothetical population. Using these hypothetical data, we reconstructed the published genetic risk models and compared their AUC values to those reported in the original articles.

**Results:** The accuracy of the AUC values varied with the method used for the construction of the risk models. When logistic regression analysis was used to construct the genetic risk model, AUC values estimated by the simulation method were similar to the published values with a median absolute difference of 0.02 [range: 0.00, 0.04]. This difference was 0.03 [range: 0.01, 0.06] and 0.05 [range: 0.01, 0.08] for unweighted and weighted risk scores.

**Conclusions:** The predictive ability of genetic risk models can be estimated using simulated data based on results from GWASs. Simulation methods can be useful to estimate the predictive ability in the absence of empirical data and to decide whether empirical investigation of genetic risk models is warranted.

## Introduction

Empirical studies on genetic risk models for multifactorial diseases so far show that the predictive ability is moderate at best (Willems et al., 2011; Husing et al., 2012), with a few promising exceptions (Maller et al., 2006; Romanos et al., 2009). The predictive ability is expected to improve further with the identification of novel genetic variants, including common variants with smaller effects and rarer variants with larger effects (Wu et al., 2011), but this improvement is not evident. For example, genetic risk models with up to 40 single-nucleotide polymorphisms (SNPs) predicted type 2 diabetes only marginally better than models with less than half of the variants included (Willems et al., 2011), and rare variants only improve the predictive ability when they are not too rare (Mihaescu et al., 2013). It can be argued that investigation of the predictive ability in empirical studies is only warranted when sufficient predictive ability is expected. This expected predictive ability may be estimated in simulation studies using hypothetical data.

Several different modeling methods have been used to investigate the predictive ability of genetic risk models (Janssens et al., 2006; Gail, 2008; Lu and Elston, 2008; Moonesinghe et al., 2010; Pepe et al., 2010). These methods all assess the predictive ability as the degree to which the risk model discriminates between patients and nonpatients, quantified as the area under the receiver operating characteristic (ROC) curve (AUC). Using epidemiological parameters such as a population-average risk of disease and the odds ratios (ORs) and frequencies of the genetic variants in the model, these methods obtain the AUC by simulating a dataset for a hypothetical population (Janssens et al., 2006; Pepe et al., 2010) or by using analytical formulas (Gail, 2008; Lu and Elston, 2008; Moonesinghe et al., 2010). A comparison of these methods showed that the simulation methods could accurately reproduce the AUC values of published prediction studies when the ORs and allele frequencies were obtained from the prediction studies themselves (Kundu et al., 2012). This observation demonstrates that the AUC value can be estimated using a simple model based on a few basic parameters.

The question that remains is whether the AUC values can also be reproduced when the ORs and frequencies are obtained from other studies or sources, such as genome-wide association studies (GWASs) that reported the variant discoveries. When that is possible, the expected predictive ability of genetic risk models can be estimated prior to the collection of empirical data to justify whether the prediction study is worth conducting. The AUC value is determined by the ORs and frequencies of the variants included in the risk model, which implies that different AUC should be expected when the ORs and frequencies differ. However, the variation in ORs and frequencies may not be large enough to produce substantially different AUCs, particularly since AUC is argued to be an insensitive metric unable to detect minor improvements in predictive ability (Cook, 2007).

To investigate how accurately AUC can be estimated in simulated data, we aimed to reproduce AUC values from published genetic prediction studies. We constructed datasets on the basis of ORs and frequencies from GWASs for the SNPs in the risk models, and compared AUC values of published prediction studies with those estimated in the simulated data. As accuracy might be related to the computational method that was used to calculate individual risks, we compared accuracy for published studies that had used unweighted or weighted risk scores or logistic regression analysis. In addition to estimating the AUC values, we explored the extent to which simulated data can reconstruct plots that are frequently presented in prediction studies.

## Materials and methods

We aimed to reproduce AUC values from published empirical prediction studies using simulated data. For each prediction study, we constructed genotypes and disease status for a hypothetical population, estimated disease risks for each hypothetical individual, and assessed the AUC of the risk model. To create a dataset for each hypothetical population, we used ORs and allele frequencies from published GWASs. The simulation method, study selection, data extraction, and analyses are described next.

### Simulation method

The simulation method created datasets of individual genotypes and disease status for hypothetical populations based on ORs and allele frequencies of genetic variants, and population-average risks of disease (Janssens et al., 2006). The datasets were constructed in such a way that the population-average disease risk, allele frequencies, and ORs estimated from the dataset match the prespecified input values. In this study, the input values were obtained from published GWASs (see below). Genotypes and disease status were constructed for 100,000 individuals. Construction of the dataset involved the following three steps, which have been described in more detail elsewhere (Janssens et al., 2006):

1. Modeling genotype data: For each SNP, the distribution of the three genotypes in the hypothetical population was based on genotype frequencies, which in turn were calculated from allele frequencies assuming Hardy-Weinberg Equilibrium. Genotypes were randomly distributed over all individuals.
2. Modeling individual disease risks: The simulation method requires disease risks to assign disease status to all individuals (step 3). Individual disease risks were estimated using Bayes' theorem, which specifies that the posterior odds of disease is obtained by multiplying the prior odds by the likelihood ratio of the individual genotype profile.
  (1) $$posterior\;odds=prior\;odds*\;\prod_{g=1}^{G}LR_{gi}$$
  where: $prior\;odds=\frac{d}{1-d}$, with *d* = population disease risk, *LR_gi_* = likelihood ratio for genotype *i* of SNP *g*.
  The likelihood ratios of genotype profiles were calculated by multiplying the likelihood ratios of the single genotypes, assuming independent effects of the SNPs (naive Bayes assumption). Posterior risks were calculated from the posterior odds using the formula: risk = odds/(1+ odds).
3. Modeling disease status: Disease status (0 or 1) was assigned based on a procedure that compares the disease risk of each individual to a randomly drawn value between 0 and 1 from a uniform distribution. An individual was assigned to develop the disease (patients) when the disease risk was higher than the random value and to not develop the disease (nonpatients) when the risk was lower than the random value.

Based on individual disease risks and disease status, the area under the receiver-operating characteristic curve (AUC) was obtained using the method of Hanley and McNeil (1982).

### Selection of genetic risk prediction studies

We aimed to reproduce empirical studies that assessed genetic risk models based on SNPs. Risk models that additionally included nongenetic risk factors or genetic variants other than SNPs, such as haplotypes and copy number variations, were not considered. We selected risk models that were unweighted risk scores, weighted risk scores or logistic regression models. Unweighted risk scores are commonly calculated as the number of risk alleles across all SNPs, assuming that all SNPs contribute equally to the risk of disease. Weighted risk scores and logistic regression models assume different effects of SNPs and calculate their cumulative effect as the sum of the log(OR) of the risk alleles across all SNPs. In weighted risk scores, the weights (ORs) are obtained from the literature, generally GWASs or meta-analyses, whereas in logistic regression models the weights are estimated in the same dataset that is used for the construction of the risk model.

To select genetic prediction studies, we searched PubMed for studies on diseases that are frequently investigated for genetic risk prediction, namely age-related macular degeneration, colorectal cancer, Crohn disease, prostate cancer, type 1 diabetes, and type 2 diabetes. For each disease we used the following search strategy in PubMed: “(genetic[title] OR genomic[title] OR genes[title] OR DNA[title] OR polymorphism[title] OR polygenic[title]) AND (risk[All Fields]) AND (score[All Fields] OR model[All Fields] OR prediction[All Fields])” (accessed August 2012). We selected studies that (1) reported the AUC value for a genetic risk model that was based on SNPs and (2) explicitly stated which SNPs were included. The PubMed search yielded 515 publications, of which 20 met the inclusion criteria. Most excluded publications were genetic association studies and studies that investigated the cumulative effect of multiple SNPs on disease risk. We additionally considered prediction studies from our recent review on type 2 diabetes (Willems et al., 2011). This review included 19 studies, of which 10 reported about genetic risk models that were based on SNPs only. Five of these were already retrieved from the PubMed search, thus a total of 25 studies were included in the present analyses.

### Data extraction

From the selected prediction studies, we retrieved citations to the GWASs, meta-analyses or pooled analyses for the SNPs included in the risk models. From these articles, we extracted unadjusted per allele ORs with the 95% confidence intervals (CIs) and the allele frequencies in controls. ORs and allele frequencies were converted, if pertinent, so that all ORs and frequencies are for risk alleles. We made the following decisions to handle multiple citations and missing data: if more than one citation was given for the same SNP, we selected the study with the largest sample size; if the cited study did not report per allele ORs, these were calculated from per genotype ORs; if CIs were not reported, these were calculated from an allele by disease status 2 × 2 contingency table using the sample size and allele frequencies from the cited study; and if allele frequencies were not reported, frequencies were obtained from the 1000 Genomes Project (1000 Genomes Project Consortium, 2012). If the cited publications did not report original analyses of OR and allele frequencies, for example when citations were reviews or earlier prediction studies, we obtained ORs and frequencies from the largest GWAS or meta-analysis published up to 12 months before the prediction study. And finally, if no GWAS or meta-analysis was published, as was the case for two SNPs in all our analysis, we used ORs and allele frequencies from the prediction study itself. Two investigators (Suman Kundu, Catherina M. C. Meijer) independently extracted data from the cited publications and discrepancies were discussed with a third investigator (Raluca Mihaescu or A. Cecile J. W. Janssens). Supplementary Table 1 lists all SNPs, risk allele frequencies and per allele ORs that were used in the analyses.

Population disease risks were obtained from the prediction studies or from epidemiological studies when disease risks were not reported. The following population disease risks were used: 20% for type 2 diabetes (Van Hoek et al., 2008), 15% for prostate cancer (Howlader et al., 2012), 0.2% for type 1 diabetes (Dabelea et al., 2014), 6.5% for age-related macular degeneration (Klein et al., 2011), 4.8% for colorectal cancer (Howlader et al., 2012), and 0.2% for Crohn disease (Kappelman et al., 2007).

### Data analyses

In the simulated data, we obtained the AUC value of the genetic risk model. When the risk model in the published prediction study was constructed as weighted risk scores or logistic regression model, Bayes theorem was used to calculate the disease risks. When the risk model was constructed as unweighted risk scores, we similarly obtained unweighted allele scores in our simulated data.

To estimate the AUC values we followed two different strategies: first, we estimated AUC using the point estimates of published ORs, and second, we randomly drew ORs from the published 95% CIs for each SNP assuming normal distribution to investigate the impact of variation in ORs on the estimated AUC values. To obtain robust estimates of the AUC, all simulations were repeated 100 times. Results are presented as averages of 100 iterations.

In addition to estimating the AUC values of the risk models, we also aimed to reproduce plots that are frequently presented in prediction studies. We selected four different plots: a histogram showing the distribution of the number of risk alleles among patients and nonpatients, a scatter plot presenting predicted risks against the number of risk alleles, a quintiles plot presenting the ORs with 95% CIs for quintiles of genetic risks, and a ROC plot showing the sensitivity vs. 1-specificity across all possible risk thresholds. For each plot we arbitrarily selected an example from the published prediction studies. We constructed the data for the hypothetical population in the same way as explained above, except that we used the sample size and population disease risk of the published prediction study because these impact the CIs in the quintiles plot and the absolute risks in the scatter plot. All analyses were performed using the PredictABEL package in R software, version 2.14.1 (www.r-project.org) (Kundu et al., 2011).

## Results

Table 1 shows the AUC estimates in the simulated data along with the published AUC values. When the prediction study had used unweighted or weighted risk scores the absolute differences between the estimated and published AUC values ranged from 0.01 to 0.06 (median: 0.03) and from 0.01 to 0.08 (median: 0.05), respectively. When prediction studies had used logistic regression models to calculate individual risks the absolute difference in AUC ranged from 0.00 to 0.04 (median: 0.02). These results were the same irrespective of whether the data were simulated on the basis of the published point estimates of the ORs or on random values from the 95% CIs (data not shown).

**Table 1:** **AUC values in published prediction studies and their estimates in simulated data**.

Published prediction study						Simulated data
Disease	First author, year	Study design	Sample size	Number of SNPs	AUC	AUC
**UNWEIGHTED RISK SCORE**						
Crohn disease	Peter et al., 2011	Case-control	872	7	0.70	0.67
Prostate cancer	Johansson et al., 2012	Case-control	1508	33	0.64^a^	0.67
Type 1 diabetes	Yamashita et al., 2011	Case-control	1743	7	0.65^a^	0.64
Type 2 diabetes	Lin et al., 2009	Cross-sectional	5360	15	0.57	0.59
Type 2 diabetes	Qi et al., 2010	Prospective cohort	3210	17	0.62	0.60
Type 2 diabetes	Van Hoek et al., 2008	Prospective cohort	6544	18	0.56	0.60
Type 2 diabetes	Meigs et al., 2008	Prospective cohort	2377	18	0.58^b^	0.59
Type 2 diabetes	Wang et al., 2010	Cross-sectional	7232	19	0.55	0.60
Type 2 diabetes	Talmud et al., 2010	Prospective cohort	5535	20	0.54	0.60
**WEIGHTED RISK SCORE**						
Prostate cancer	Sun et al., 2011	Case-control	4621	28	0.62	0.66
Prostate cancer	Kader et al., 2012	Case-control	1654	33	0.59	0.67
Type 2 diabetes	Lin et al., 2009	Cross-sectional	5360	15	0.59	0.60
Type 2 diabetes	Talmud et al., 2010	Prospective cohort	5535	20	0.55	0.61
**LOGISTIC REGRESSION MODEL**						
AMD	Scholl et al., 2008	Case-control	179	3	0.73	0.69
AMD	Hecker et al., 2010	Case-control	274	4	0.77	0.76
AMD	Grassmann et al., 2012	Case-control	1782	13	0.82	0.78
Colorectal cancer	Dunlop et al., 2013	Case-control	39266	10	0.57	0.60
Colorectal cancer	Lubbe et al., 2012	Prospective cohort	14929	14	0.58	0.60
Crohn disease	Peter et al., 2011	Case-control	872	7	0.71	0.68
Prostate cancer	Aly et al., 2011	Prospective cohort	5241	36	0.67	0.69
Prostate cancer	Helfand et al., 2010	Case-control	1464	9	0.61	0.62
Type 2 diabetes	Weedon et al., 2006	Case-control	6077	3	0.58	0.59
Type 2 diabetes	Vaxillaire et al., 2008	Prospective cohort	5212	3	0.56	0.58
Type 2 diabetes	Hu et al., 2009	Case-control	3634	11	0.62	0.61
Type 2 diabetes	Miyake et al., 2009	Case-control	2000	11	0.63	0.63
Type 2 diabetes	Fontaine-Bisson et al., 2010	Cross-sectional	2751	17	0.59	0.61
Type 2 diabetes	Van Hoek et al., 2008	Prospective cohort	6544	18	0.60	0.61
Type 2 diabetes	Lango et al., 2008	Case-control	4907	18	0.60	0.61
Type 2 diabetes	Sparso et al., 2009	Case-control	9395	19	0.60	0.61

AUC, area under the receiver operating characteristic curve; CI, confidence interval; AMD, age-related macular degeneration; SNP, single nucleotide polymorphism.

a Adjusted for age;

b *Adjusted for sex*.

To illustrate that minor differences in ORs and risk allele frequencies may not impact AUC, we present the ORs of two different studies (Lango et al., 2008; Van Hoek et al., 2008) that investigated the same risk model for the prediction of type 2 diabetes (Table 2). These studies showed the same AUC value (0.60), but the individual SNPs had different ORs. For example, the OR of *KCNJ11* was 1.25 in the GoDARTS study and 1.03 in the Rotterdam study, and the OR of *NOTCH2* was 1.15 and 1.01, respectively. The ORs from the GWASs were similar for the two studies, but generally higher than those reported in the two prediction studies themselves. Risk allele frequencies did not markedly differ between the cited studies and the prediction studies (Supplementary Table 2). The estimated AUC values in simulated data were 0.61 for both studies.

**Table 2:** **Odds ratios of 18 single nucleotide polymorphisms in two prediction studies on type 2 diabetes and their corresponding values in the cited genome-wide association studies**.

Gene	SNP	OR in prediction study		OR in cited GWAS^*^	
		GoDARTS study	Rotterdam study	GoDARTS study	Rotterdam study
*ADAM30/NOTCH2*	rs2641348^†^	1.15 (1.01, 1.30)	1.01 (0.88, 1.17)	1.10 (1.06, 1.15)	
*ADAMTS9*	rs4607103^‡‡^	1.05 (0.96, 1.16)	1.14 (1.03, 1.28)	1.09 (1.06, 1.12)	
*CDC123*	rs12779790^¥^	1.10 (0.99, 1.21)	1.05 (0.94, 1.19)	1.11 (1.07, 1.14)	
*CDKAL1*	rs10946398^§^	1.11 (1.02, 1.21)	1.11 (1.02, 1.22)	1.12 (1.08, 1.16)^1^	
*CDKN2A/2B*	rs10811661	1.21 (1.08, 1.35)	1.10 (0.98, 1.24)	1.20 (1.14, 1.25)	
*CDKN2A/2B*	rs564398^‡^	1.13 (1.04, 1.22)	1.04 (0.95, 1.14)	1.12 (1.07, 1.17)	
*FTO*	rs8050136	1.11 (1.02, 1.20)	1.09 (0.99, 1.19)	1.15 (1.09, 1.22)	
*HHEX-IDE*	rs1111875	1.02 (0.94, 1.11)	1.06 (0.97, 1.15)	1.13 (1.08, 1.17)	
*IGF2BP2*	rs4402960	1.12 (1.03, 1.22)	1.11 (1.01, 1.22)	1.17 (1.10, 1.25)	
*JAZF1*	rs864745^§§^	1.00 (0.93, 1.09)	1.09 (1.00, 1.19)	1.10 (1.07, 1.13)	
*KCNJ11*	rs5219	1.25 (1.15, 1.36)	1.03 (0.93, 1.13)	1.18 (1.04, 1.34)	1.14 (1.10, 1.19)
*PPARG*	rs1801282	1.21 (1.07, 1.36)	1.09 (0.95, 1.24)	1.14 (1.08, 1.20)	
*SLC30A8*	rs13266634	1.10 (1.01, 1.20)	1.13 (1.02, 1.24)	1.12 (1.07, 1.16)	
*TCF2*	rs757210^††^	1.07 (0.99, 1.16)	1.07 (0.98, 1.18)	1.12 (1.07, 1.18)	1.22 (1.15, 1.30)^1^
*TCF7L2*	rs7903146	1.36 (1.24, 1.48)	1.31 (1.19, 1.44)	1.47 (1.33, 1.62)	1.38 (1.31, 1.46)
*THADA*	rs7578597	1.04 (0.90, 1.19)	1.10 (0.96, 1.27)	1.15 (1.10, 1.20)	
*TSPAN8/LGR5*	rs7961581^¶^	1.09 (1.00, 1.19)	1.09 (0.99, 1.20)	1.09 (1.06, 1.12)	
*WFS1*	rs10010131^**^	1.07 (0.99, 1.16)	1.12 (1.05, 1.27)	1.11 (1.07, 1.16)	

Table is adapted from Janssens and Van Duijn (2009). The risk models of the GoDARTS study (Lango et al., 2008) and the Rotterdam Study (Van Hoek et al., 2008) included the same 18 genes and both had an AUC of 0.60. The AUC values from simulated data were the same and both were 0.61. The SNPs listed in the table are those used by the GoDARTS study. For several genes, the Rotterdam Study used different SNPs that were in linkage disequilibrium:

† rs1493694, r^2^ = 0.74;

‡ rs1412829, r^2^ = 0.97;

§ rs7754840, r^2^ = 1.00;

¶ rs1353362, r^2^ = 0.96;

¥ rs11257622; r^2^ = 0.83;

** rs10012946, r^2^ = 1.00;

†† rs4430796, r^2^ = 0.61;

‡‡ rs4411878, r^2^ = 0.95;

§§ rs1635852, r^2^ = 0.97. SNP, single nucleotide polymorphism; GWAS, genome-wide association study.

* When only one value is presented, both prediction studies cited the same GWAS.

1 *GWAS studies reported for the SNP used by the Rotterdam study; all others are for SNPs used by the GoDARTS study*.

**Figure 1:**
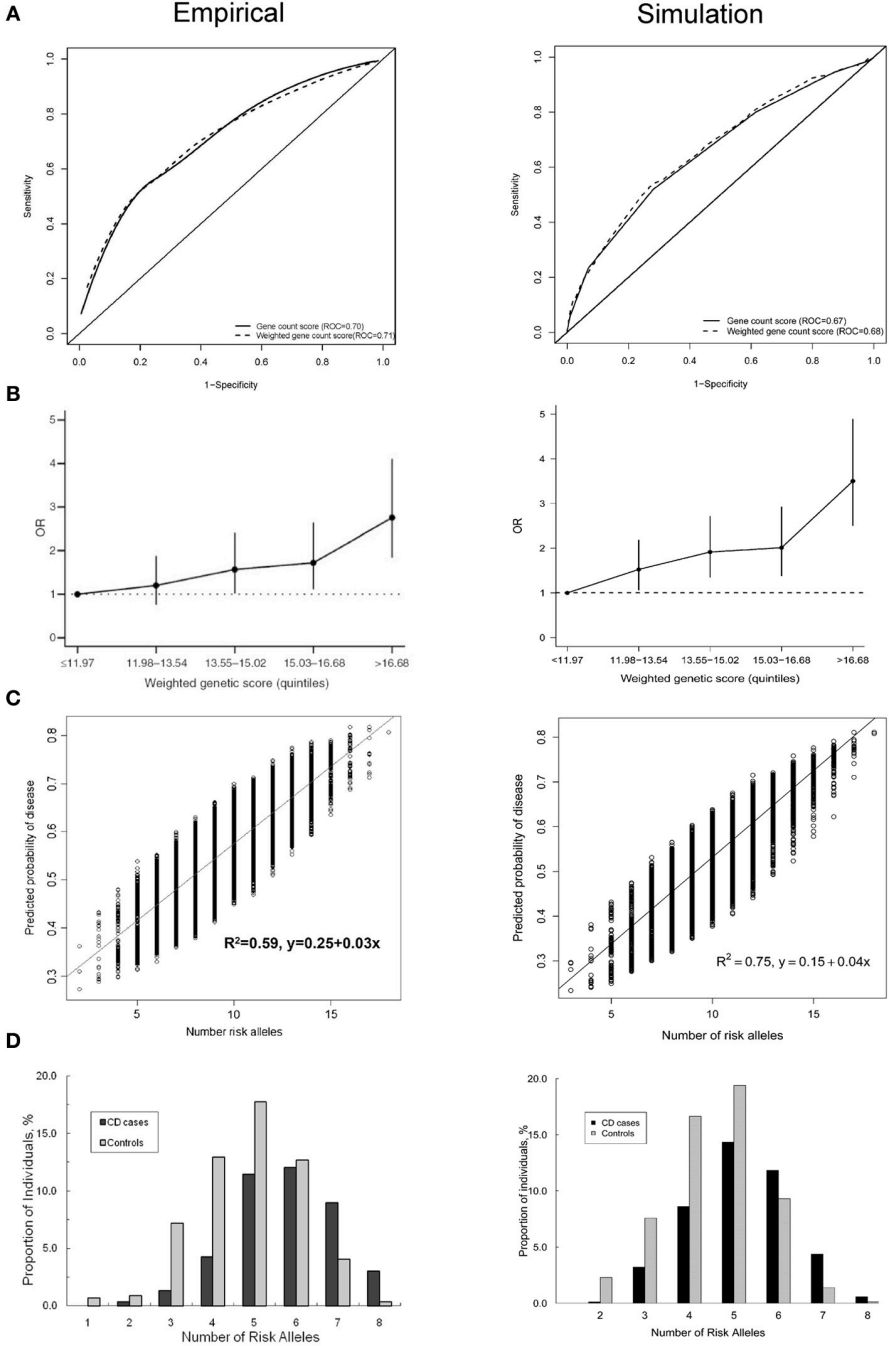
**Plots published in empirical prediction studies and their reproductions in simulated data. (A)** Receiver operating characteristic (ROC) curves for unweighted and weighted gene count scores (Peter et al., 2011). **(B)** Quintiles plot presenting the odds ratios with 95% confidence intervals by quintiles of the weighted genetic scores (Lin et al., 2009). **(C)** Scatter plot showing the variation in predicted risks stratified by the number of risk alleles (Dunlop et al., 2013). **(D)** Histogram showing the distribution of the number of risk alleles among patients and nonpatients (Peter et al., 2011).

Figure 1 shows four plots produced by the simulation method and their original versions in the published simulation studies. The ROC curve and the histogram were relatively similar between the simulation and published studies (Figures 1A,D), but the quintiles and scatter plots showed larger differences (Figures 1B,C). The scatterplot showed a similar spread of predicted risks for each number of risk alleles in both the empirical and simulation study, but the *R*^2^-value was higher for the simulated data. For the quintiles plot, the accuracy of the reproduction was affected by the choice of risk thresholds that define the quintiles and by the simulation sample size. In separate iterations, the graphs in the simulated data differed most when the sample size and risk thresholds of the published study were used (Supplementary Figure 1A). The graphs were markedly similar when the risk thresholds were chosen based on a quintile distribution in the simulation study (Supplementary Figure 1B), or when sample size was increased to 100,000 (Supplementary Figure 1C). When the simulated dataset had large sample size, the estimated ORs for the quintiles were very similar with that in the published study, but the confidence intervals were narrower.

## Discussion

We investigated how accurately simulation studies can estimate the AUC values from empirical genetic prediction studies using ORs and frequencies from GWASs. The simulation method used in this study could reproduce AUC values fairly accurately, predominantly when prediction studies used logistic regression models to obtain individual risks. The simulation method could also reproduce plots that are frequently reported in prediction studies.

Before discussing the implications of our findings, the assumptions of the simulation method need to be addressed. To estimate individual disease risks, the method assumes that (1) the combined effect of genetic variants follows a multiplicative (log additive) risk model; (2) genetic variants inherit independently; (3) genetic variants have independent effects on the disease risk; and (4) effect sizes for genetic variants are considered as unadjusted per allele ORs (marginal effects). These assumptions may impact the predictive ability of risk models and therefore affect AUC values (Moonesinghe et al., 2011), but they appear to be valid for two reasons. First, these assumptions are also considered in empirical prediction studies, with the exception of the marginal effect sizes. Both our approach, based on Bayes theorem, and the weighted risk scores are based on marginal effect sizes of the SNPs, but in logistic regression models the effect sizes of the SNPs are simultaneously estimated, adjusted for each other. Yet, the difference between marginal and adjusted effect sizes seems not large enough to affect an aggregate measure like AUC, as was also observed by others (Wu et al., 2013). Second, we recently showed that observed AUC values and those estimated from simulated data were similar when ORs and frequencies of the genetic variants were obtained from the empirical prediction study itself (Kundu et al., 2012), suggesting that the modeling method itself produces accurate results. Therefore, we do not expect that the modeling assumptions and the simulation method as such have influenced the estimated values of AUC.

Our analyses focused on risks scores and logistic regression models because these are most frequently used in empirical prediction research. One might argue that our simulation method might soon become outdated because these models are very simple, but more sophisticated risk models do not evidently show higher predictive ability. Risk models based on neural networks, decision trees, support vector machines (Forberg et al., 2009; Gulkesen et al., 2010; Lee et al., 2010; Muniz et al., 2010; Wu et al., 2010; Kim et al., 2011; Van Der Ploeg et al., 2011) often show higher predictive ability than logistic regression models in data that were used to develop the models (Lee et al., 2010; Muniz et al., 2010; Kim et al., 2011), but are frequently outperformed by logistic regression analyses in validation studies (Forberg et al., 2009; Gulkesen et al., 2010; Wu et al., 2010; Van Der Ploeg et al., 2011). This suggests that logistic regression is expected to remain relevant for constructing genetic risk models in the future.

Our study showed that the AUC value of genetic risk prediction models can be estimated from the ORs and allele frequencies from GWASs. Estimated AUCs generally approximated the published AUCs because the ORs and allele frequencies from GWASs did not markedly differ from those observed in the prediction studies. Typically, some odds ratios and frequencies were higher and others were lower, so on average they resulted in a similar AUC as published.

The AUC values estimated in simulated data approximated the published AUCs, but a detailed look into the discrepancies shows that the simulation method tends to overestimate rather than underestimate the AUC values. We do expect the slight overestimation, because GWAS results by definition are selected on the basis of their high OR in the GWAS. Independent investigation of these SNP effects, such as in empirical prediction studies, is more likely to show lower than higher ORs. When the simulation method uses the higher GWAS ORs, a slightly higher AUC should be expected.

While most AUC values tend to be slightly overestimated by the simulation method, several others were underestimated. Table 1 shows that we underestimated the AUC values of prediction studies with smaller sample size. This might be a consequence of publication bias. Prediction studies with small sample size might only be published when they show higher predictive ability. If so, the contributing ORs must be high as well, and likely be higher than the ORs from GWAS. This is indeed what we observed. The ORs of two major SNPs in the AMD studies (Scholl et al., 2008; Grassmann et al., 2012) were markedly higher than those in the GWAS, and hence led to higher AUC than what we estimated on the basis of the GWAS results in the simulated data. This observation underscores the importance of sufficient sample size in empirical prediction studies to prevent overestimation of predictive ability.

AUC values were more accurate for empirical studies that used logistic regression models than for studies that investigated weighted risk scores. This difference might be explained by differences in model fit. When logistic regression models are used to estimate individual risks, the ORs of the variants in the model reflect the “true” ORs in the population under study, but this is not the case when weighted risk scores are calculated. In empirical studies that calculate weighted risk scores, the weights are obtained from GWASs and meta-analyses, and these usually differ from the ORs observed in the study itself. Yet, in our modeling the ORs to construct the hypothetical dataset and the weighted risk scores were the same. Therefore, the weighted risk scores by definition have better model fit in simulated data, which might explain the higher AUC estimates.

Simulation studies can be used to assess the predictive ability when empirical data are not available and their collection is not an option. We recently investigated the predictive ability of personal genome tests that were offered by three companies directly to consumers via the internet. Using the simulation method described in this study, we showed for six diseases that the predictive ability on the population level was similar between the companies, but that for individual consumers differences in predicted risks were substantial (Kalf et al., 2014). The simulation method was able to provide insight in the predictive ability of commercial genome tests that would otherwise not have been available.

Simulation methods that investigate the predictive ability of genetic risk models can also be used to obtain the expected predictive performance of genetic variants before conducting empirical studies. The method can be used to justify whether empirical assessment of the risk models is warranted or, in case the expected predictive ability is not promising, whether further genomic discoveries should be awaited.

## Author contributions

A. Cecile J. W. Janssens conceived and supervised the study and designed the initial version of the simulation model; Suman Kundu and Catherina M. C. Meijer conducted the data extraction, under supervision of Raluca Mihaescu and A. Cecile J. W. Janssens. Suman Kundu programmed the simulation model, performed the statistical analyses, and drafted the first version of the manuscript. Suman Kundu, Raluca Mihaescu, Rachel Bakker, and A. Cecile J. W. Janssens revised the manuscript. All authors read and approved the final version of the manuscript.

### Conflict of interest statement

The authors declare that the research was conducted in the absence of any commercial or financial relationships that could be construed as a potential conflict of interest.

## Supplementary material

The supplementary material includes Supplementary Table 1 with details about the single-nucleotide polymorphisms (SNPs) considered in the analyses, Supplementary Table 2 with allele frequencies for SNPs from two prediction studies and their corresponding values in the cited genome-wide association studies, and Supplementary Figure 1 with quintile plots presenting the odds ratios for quintiles of genetic risk scores.
