# Supplementary material for: Estimating the predictive ability of genetic risk models in simulated data based on published results from genome-wide association studies
Source: Front Genet. 2014 Jun 13;5:179. doi: 10.3389/fgene.2014.00179 (PMC4056181; doi:10.3389/fgene.2014.00179)
Supplement: Supplementary file 1 [file DataSheet1.DOC]

***Supplementary material***

**Estimating the predictive ability of genetic risk models in simulated data based on published results from genome-wide association studies**

**Suman Kundu^1^, Raluca Mihaescu^1^, Catherina M.C. Meijer^1^, Rachel Bakker^1^, A. Cecile J.W. Janssens^1,2*^**

^1^Department of Epidemiology, Erasmus University Medical Center, 3015 GE Rotterdam, the Netherlands; ^2^Department of Epidemiology, Rollins School of Public Health, Emory University, Atlanta, Georgia 30322, USA

^*^**Correspondence:**

Professor A. Cecile J. W. Janssens, Department of Epidemiology, Rollins School of Public Health, Emory University, 1518 Clifton Road NE, Atlanta, Georgia 30322, USA.

cecile.janssens@emory.edu

**Supplementary Table 1** Risk allele frequencies and odds ratios of single-nucleotide polymorphisms considered in the analyses

Legend: Locus of each SNP was obtained from Ensembl database release 68 . Gene names were obtained from the cited publication when these names were the same as in the Ensembl database release 68 or the GWAS catalog (accessed June 2013) . Alternatively, we used, in subsequent order, the gene name reported in the GWAS catalog, the cited publication, or the prediction study; otherwise the SNP was denoted as ‘intergenic’. Locus information was obtained from the Ensembl database. Risk alleles, risk allele frequencies and odds ratios were obtained from the cited publications, unless otherwise indicated.

SNP, single nucleotide polymorphism; OR, odds ratio; CI, confidence interval.

* Risk allele frequency obtained from 1000 genomes project .

A. Age-related macular degeneration

| **SNP** | **Locus** | **Gene** | **Risk allele** | **Risk allele frequency** | **OR (95% CI)** | **Reference** |
| --- | --- | --- | --- | --- | --- | --- |
| rs1329424 | 1q31.3 | *CFH* | T | 0.35 | 1.88 (1.68 - 2.10) |  |
| rs6677604 | 1q31.3 | *CFH* | G | 0.86 | 2.85 (1.77 - 4.58) |  |
| rs2274700 | 1q31.3 | *HF1/CFH* | G | 0.68 | 2.39 (1.98 - 2.89) |  |
| rs800292 | 1q31.3 | *HF1/CFH* | G | 0.37 | 1.13 (0.96 - 1.34) |  |
| rs2285714 | 4q25 | *PLA2G12A* | T | 0.40 | 1.31 (1.18 - 1.45) |  |
| rs4151667 | 6p21.33 | *BF* | T | 0.95* | 2.78 (1.79 - 4.35) |  |
| rs547154 | 6p21.33 | *C2* | G | 0.91* | 2.27 (1.67 - 3.03) |  |
| rs10490924 | 10q26.13 | *ARMS2* | T | 0.21 | 3.00 (2.64 - 3.40) |  |
| rs10490924 | 10q26.13 | *ARMS2* | T | 0.33 | 2.97 (2.58 - 3.43) |  |
| rs10468017 | 15q21.3 | *RPL28P4 - LIPC* | C | 0.70 | 1.22 (1.14 - 1.30) |  |
| rs493258 | 15q21.3 | *RPL28P4 - LIPC* | C | 0.56 | 1.14 (1.09 - 1.20) |  |
| rs2230199 | 19p13.3 | *C3* | C | 0.16 | 1.74 (1.47 - 2.06) |  |
| rs2230199 | 19p13.3 | *C3* | G | 0.26 | 1.66 (1.44 - 1.92) |  |
| rs429358 | 19q13.32 | *APOE* | C | 0.89 | 1.28 (1.01 - 1.64) |  |
| rs7412 | 19q13.32 | *APOE* | C | 0.09 | 1.55 (1.17 - 2.07) |  |
| rs9621532 | 22q12.3 | *SYN3* | A | 0.95 | 1.41 (1.27 - 1.57) |  |

B. Colorectal cancer

| **SNP** | **Locus** | **Gene** | **Risk allele** | **Risk allele frequency** | **OR (95% CI)** | **Reference** |
| --- | --- | --- | --- | --- | --- | --- |
| rs6691170 | 1q41 | *DUSP10 - QRSL1P2* | T | 0.40* | 1.06 (1.03 - 1.09) |  |
| rs10936599 | 3q26.2 | *MYNN* | C | 0.75* | 1.08 (1.04 - 1.10) |  |
| rs16892766 | 8q23.3 | *LINC00536 - EIF3H* | A | 0.07 | 1.25 (1.19 - 1.32) |  |
| rs6983267 | 8q24.21 | *SRRM1P1 - POU5F1B* | G | 0.48* | 1.21 (1.15 - 1.27) |  |
| rs10795668 | 10p14 | *CHCHD3P1 - HSP90AB7P* | A | 0.67 | 1.12 (1.10 - 1.16) |  |
| rs3802842 | 11q23.1 | *C11orf93; C11orf92* | C | 0.43 | 1.11 (1.08 - 1.15) |  |
| rs11169552 | 12q13.12 | *DIP2B - ATF1* | C | 0.75* | 1.09 (1.05 - 1.11) |  |
| rs4444235 | 14q22.2 | *BMP4* | C | 0.46 | 1.11 (1.08 - 1.15) |  |
| rs4779584 | 15q13.3 | *SCG5 - GREM1* | T | 0.76 | 1.54 (1.46 - 1.62) |  |
| rs9929218 | 16q22.1 | *CDH1* | G | 0.71 | 1.10 (1.06 - 1.12) |  |
| rs4939827 | 18q21.1 | *SMAD7* | T | 0.48 | 1.18 (1.12 - 1.23) |  |
| rs4939827 | 18q21.1 | *SMAD7* | T | 0.52 | 1.20 (1.16 - 1.24) |  |
| rs10411210 | 19q13.11 | *RHPN2* | C | 0.90 | 1.15 (1.10 - 1.20) |  |
| rs961253 | 20p12.3 | *FGFR3P3 - BMP2* | A | 0.36 | 1.12 (1.08 - 1.16) |  |
| rs4925386 | 20q13.33 | *LAMA5* | C | 0.68* | 1.08 (1.05 - 1.10) |  |

C. Crohn's disease

| **SNP** | **Locus** | **Gene** | **Risk allele** | **Risk allele frequency** | **OR (95% CI)** | **Reference** |
| --- | --- | --- | --- | --- | --- | --- |
| rs11209026 | 1p31.3 | *IL23R* | G | 0.93 | 2.22 (1.37 - 3.70) |  |
| rs7517847 | 1p31.3 | *IL23R* | T | 0.65 | 1.72 (1.39 - 2.13) |  |
| rs1373692 | 5p13.1 | *INTS6P1 - PTGER4* | G | 0.64 | 1.45 (1.26 - 1.68) |  |
| rs13361189 | 5q33.1 | *IRGM* | C | 0.08 | 1.38 (1.15 - 1.66) |  |
| rs2066844 | 16q12.1 | *NOD2* | T | 0.04 | 1.74 (0.88 - 3.42) |  |
| rs2066845 | 16q12.1 | *NOD2* | C | 0.05 | 1.93 (1.23 - 3.00) |  |
| rs2066847 | 16q12.1 | *NOD2* | C | 0.03 | 2.45 (1.51 - 3.98) |  |

D. Prostate cancer

| **SNP** | **Locus** | **Gene** | **Risk allele** | **Risk allele frequency** | **OR (95% CI)** | **Reference** |
| --- | --- | --- | --- | --- | --- | --- |
| rs2710646 | 2p15 | *EHBP1* | A | 0.20 | 1.16 (1.06 - 1.26) |  |
| rs2710647 | 2p15 | *EHBP1* | C | 0.56 | 1.11 (0.95 - 1.30) |  |
| rs721048 | 2p15 | *EHBP1* | A | 0.19 | 1.15 (1.10 - 1.21) |  |
| rs721048 | 2p15 | *EHBP1* | A | 0.14 | 1.18 (1.10 - 1.28) |  |
| rs1465618 | 2p21 | *THADA* | A | 0.23 | 1.08 (1.03 - 1.12) |  |
| rs1465618 | 2p21 | *THADA* | A | 0.21 | 1.15 (1.04 - 1.26) |  |
| rs12621278 | 2q31.1 | *ITGA6* | A | 0.94 | 1.33 (1.25 - 1.43) |  |
| rs12621278 | 2q31.1 | *ITGA6* | A | 0.96 | 1.35 (1.27 - 1.44) |  |
| rs2660753 | 3p12.1 | *PPATP1 - MIR4795* | T | 0.11 | 1.19 (1.09 - 1.31) |  |
| rs2660753 | 3p12.1 | *PPATP1 - MIR4795* | T | 0.10 | 1.24 (1.04 - 1.48) |  |
| rs10934853 | 3q21.3 | *EEFSEC* | A | 0.28 | 1.12 (1.08 - 1.16) |  |
| rs10934853 | 3q21.3 | *EEFSEC* | A | 0.24 | 1.12 (1.06 - 1.18) |  |
| rs4857841 | 3q21.3 | *EEFSEC* | A | 0.28 | 1.12 (1.08 - 1.16) |  |
| rs12500426 | 4q22.3 | *PDLIM5* | A | 0.46 | 1.08 (1.05 - 1.12) |  |
| rs17021918 | 4q22.3 | *PDLIM5* | C | 0.66 | 1.11 (1.08 - 1.15) |  |
| rs17021918 | 4q22.3 | *PDLIM5* | C | 0.65 | 1.14 (1.10 - 1.18) |  |
| rs7679673 | 4q24 | *TET2* | C | 0.55 | 1.10 (1.06 - 1.14) |  |
| rs7679673 | 4q24 | *TET2* | C | 0.62 | 1.14 (1.09 - 1.20) |  |
| rs9364554 | 6q25.3 | *SLC22A3* | T | 0.31 | 1.21 (1.14 - 1.28) |  |
| rs9364554 | 6q25.3 | *SLC22A3* | T | 0.27 | 1.17 (1.06 - 1.29) |  |
| rs10486567 | 7p15.2 | *JAZF1* | G | 0.77 | 1.11 (1.09 - 1.13) |  |
| rs12155172 | 7p15.3 | *RPS26P30 - ASS1P11* | A | 0.20 | 1.05 (1.0 - 1.10) |  |
| rs6465657 | 7q21.3 | *LMTK2* | C | 0.48 | 1.18 (1.12 - 1.25) |  |
| rs6465657 | 7q21.3 | *LMTK2* | C | 0.51 | 1.14 (1.05 - 1.23) |  |
| rs1512268 | 8p21.2 | *FAM60DP - NKX3.1* | A | 0.45 | 1.18 (1.14 - 1.22) |  |
| rs1512268 | 8p21.2 | *FAM60DP - NKX3.1* | T | 0.42 | 1.17 (1.12 - 1.23) |  |
| rs2928679 | 8p21.2 | *NKX3.1* | T | 0.42 | 1.05 (1.01 - 1.09) |  |
| rs2928679 | 8p21.2 | *NKX3.1* | A | 0.46 | 1.13 (1.02 - 1.25) |  |
| rs4961199 | 8q21.3 | *CPNE3* | A | 0.16 | 1.17 (1.15 - 1.20) |  |
| rs620861 | 8q24.21 | *Intergenic* | G | 0.62 | 1.16 (1.11 - 1.20) |  |
| rs1447295 | 8q24.21 | *LOC727677* | A | 0.09 | 1.60 (1.43 - 1.77) |  |
| rs1447295 | 8q24.21 | *LOC727677* | A | 0.11 | 1.58 (1.43 - 1.74) |  |
| rs1447295 | 8q24.21 | *LOC727677* | A | 0.07 | 1.47 (1.33 - 1.62) |  |
| rs10086908 | 8q24.21 | *NKX3.1* | T | 0.72 | 1.15 (1.08 - 1.22) |  |
| rs10086908 | 8q24.21 | *NKX3.1* | T | 0.63 | 1.13 (1.08 - 1.19) |  |
| rs12543663 | 8q24.21 | *NKX3.1* | C | 0.32 | 1.13 (1.07 - 1.20) |  |
| rs620861 | 8q24.21 | *NKX3.1* | T | 0.64 | 1.17 (1.11 - 1.23) |  |
| rs1016343 | 8q24.21 | *PCAT1 - SRRM1P1* | T | 0.22 | 1.26 (1.18 - 1.34) |  |
| rs1016343 | 8q24.21 | *PCAT1 - SRRM1P1* | T | 0.21 | 1.39 (1.24 - 1.55) |  |
| rs13252298 | 8q24.21 | *PCAT1 - SRRM1P1* | G | 0.72 | 1.19 (1.13 - 1.26) |  |
| rs4242382 | 8q24.21 | *POU5F1B - MYC* | A | 0.12 | 1.46 (1.42 - 1.50) |  |
| rs16901979 | 8q24.21 | *SRRM1P1 - POU5F1B* | A | 0.03 | 1.79 (1.53 - 2.11) |  |
| rs16901979 | 8q24.21 | *SRRM1P1 - POU5F1B* | A | 0.04 | 1.80 (1.55 - 2.09) |  |
| rs16901979 | 8q24.21 | *SRRM1P1 - POU5F1B* | A | 0.03 | 1.82 (1.44 - 2.30) |  |
| rs16902094 | 8q24.21 | *SRRM1P1 - POU5F1B* | G | 0.15 | 1.21 (1.15 - 1.26) |  |
| rs16902094 | 8q24.21 | *SRRM1P1 - POU5F1B* | G | 0.27 | 1.20 (1.12 - 1.30) |  |
| rs445114 | 8q24.21 | *SRRM1P1 - POU5F1B* | T | 0.64 | 1.14 (1.10 - 1.19) |  |
| rs6983267 | 8q24.21 | *SRRM1P1 - POU5F1B* | G | 0.54 | 1.31 (1.24 - 1.38) |  |
| rs6983267 | 8q24.21 | *SRRM1P1 - POU5F1B* | G | 0.53 | 1.41 (1.29 - 1.54) |  |
| rs6983267 | 8q24.21 | *SRRM1P1 - POU5F1B* | G | 0.49 | 1.20 (1.14 - 1.26) |  |
| rs6983267 | 8q24.21 | *SRRM1P1 - POU5F1B* | G | 0.53 | 1.24 (1.22 - 1.27) |  |
| rs6983267 | 8q24.21 | *SRRM1P1 - POU5F1B* | G | 0.53 | 1.26 (1.19 - 1.34) |  |
| rs6983561 | 8q24.21 | *SRRM1P1 - POU5F1B* | C | 0.04 | 1.66 (1.43 - 1.92) |  |
| rs1571801 | 9q33.2 | *DAB2IP* | A | 0.25 | 1.28 (1.17 - 1.40) |  |
| rs10993994 | 10q11.23 | *MSMB* | T | 0.42 | 1.37 (1.30 - 1.45) |  |
| rs10993994 | 10q11.23 | *MSMB* | T | 0.34 | 1.25 (1.12 - 1.40) |  |
| rs10993994 | 10q11.23 | *MSMB* | T | 0.41 | 1.28 (1.25 - 1.31) |  |
| rs10993994 | 10q11.23 | *MSMB* | T | 0.40 | 1.26 (1.24 - 1.28) |  |
| rs4962416 | 10q26.13 | *CTBP2* | C | 0.26 | 1.15 (1.04 - 1.27) |  |
| rs4962416 | 10q26.13 | *CTBP2* | C | 0.27 | 1.20 (1.17 - 1.22) |  |
| rs7127900 | 11p15.5 | *MIR4686 - ASCL2* | A | 0.20 | 1.22 (1.17 - 1.27) |  |
| rs7127900 | 11p15.5 | *MIR4686 - ASCL2* | A | 0.24 | 1.25 (1.20 - 1.30) |  |
| rs12418451 | 11q13.3 | *CTBP2* | A | 0.30 | 1.16 (1.09 - 1.23) |  |
| rs10896450 | 11q13.3 | *Intergenic* | G | 0.47 | 1.13 (1.06 - 1.21) |  |
| rs10896449 | 11q13.3 | *MIR3164 - MYEOV* | G | 0.53 | 1.28 (1.16 - 1.40) |  |
| rs10896449 | 11q13.3 | *MIR3164 - MYEOV* | G | 0.53 | 1.16 (1.11 - 1.22) |  |
| rs10896449 | 11q13.3 | *MIR3164 - MYEOV* | G | 0.52 | 1.22 (1.19 - 1.24) |  |
| rs11228565 | 11q13.3 | *MIR3164 - MYEOV* | A | 0.20 | 1.23 (1.16 - 1.31) |  |
| rs11649743 | 17q12 | *HNF1B* | G | 0.76 | 1.16 (1.11 - 1.22) |  |
| rs11649743 | 17q12 | *HNF1B* | G | 0.82 | 1.18 (1.11 - 1.24) |  |
| rs4430796 | 17q12 | *HNF1B* | A | 0.49 | 1.22 (1.15 - 1.30) |  |
| rs4430796 | 17q12 | *HNF1B* | A | 0.49 | 1.22 (1.17 - 1.26) |  |
| rs7501939 | 17q12 | *HNF1B* | T | 0.61 | 1.41 (1.29 - 1.55) |  |
| rs1859962 | 17q24.3 | *CALM2P1 - SOX9* | G | 0.49 | 1.27 (1.16 - 1.40) |  |
| rs1859962 | 17q24.3 | *CALM2P1 - SOX9* | G | 0.46 | 1.20 (1.14 - 1.27) |  |
| rs1859962 | 17q24.3 | *CALM2P1 - SOX9* | G | 0.53 | 1.21 (1.12 - 1.30) |  |
| rs887391 | 19a13.2 | *Intergenic* | T | 0.76 | 1.14 (1.08 - 1.20) |  |
| rs8102476 | 19q13.2 | *DPF1 - PPP1R14A* | C | 0.54 | 1.12 (1.08 - 1.15) |  |
| rs8102476 | 19q13.2 | *DPF1 - PPP1R14A* | C | 0.50 | 1.12 (1.08 - 1.15) |  |
| rs266849 | 19q13.33 | *KLK15 - KLK3* | G | 0.80 | 1.24 (1.16 - 1.33) |  |
| rs2735839 | 19q13.33 | *KLK3 - KLK2* | A | 0.85 | 1.42 (1.31 - 1.53) |  |
| rs2735839 | 19q13.33 | *KLK3 - KLK2* | G | 0.86 | 1.30 (1.11 - 1.51) |  |
| rs9623117 | 22q13.1 | *TNRC6B* | C | 0.22 | 1.13 (1.05 - 1.22) |  |
| rs9623117 | 22q13.1 | *TNRC6B* | C | 0.21 | 1.18 (1.11 - 1.26) |  |
| rs5759167 | 22q13.2 | *RPS25P10 - BIK* | G | 0.53 | 1.16 (1.14 - 1.20) |  |
| rs5759167 | 22q13.2 | *RPS25P10 - BIK* | G | 0.55 | 1.18 (1.14 - 1.21) |  |
| rs5945572 | Xp11.22 | *NUDT10 - NUDT11* | A | 0.35 | 1.23 (1.16 - 1.30) |  |
| rs5945619 | Xp11.22 | *NUDT11 - CENPVP3* | C | 0.38 | 1.28 (1.21 - 1.35) |  |
| rs5945619 | Xp11.22 | *NUDT11 - CENPVP3* | C | 0.39 | 1.27 (1.12 - 1.43) |  |

E. Type 1 diabetes

| **SNP** | **Locus** | **Gene** | **Risk allele** | **Risk allele frequency** | **OR (95% CI)** | **Reference** |
| --- | --- | --- | --- | --- | --- | --- |
| rs1990760 | 2q24.2 | *IFIH1* | A | 0.61 | 1.18 (1.06 - 1.19) |  |
| rs3087243 | 2q33.2 | *CTLA4* | G | 0.75 | 1.04 (0.88 - 1.23) |  |
| rs6897932 | 5p13.2 | *IL7R* | G | 0.73 | 1.12 (1.06 - 1.19) |  |
| rs706778 | 10p15.1 | *IL2RA* | A | 0.54 | 1.23 (1.06 - 1.43) |  |
| rs689 | 11p15.5 | *INS* | T | 0.67* | 3.29 (1.68 - 6.43) |  |
| rs2292239 | 12q13.2 | *ERBB3* | A | 0.27 | 1.38 (1.16 - 1.65) |  |
| rs2903692 | 16p13.13 | *CLEC16A* | G | 0.85 | 1.28 (1.05 - 1.57) |  |

F. Type 2 diabetes

| **SNP** | **Locus** | **Gene** | **Risk allele** | **Risk allele frequency** | **OR (95% CI)** | **Reference** |
| --- | --- | --- | --- | --- | --- | --- |
| rs10923931 | 1p12 | *NOTCH2* | T | 0.11 | 1.13 (1.08 - 1.17) |  |
| rs2641348 | 1p31.1 | *ADAM30* | G | 0.11 | 1.10 (1.06 - 1.15) |  |
| rs10490072 | 2p16.1 | *BCL11A* | T | 0.72 | 1.05 (1.03 - 1.08) |  |
| rs7578597 | 2p21 | *THADA* | T | 0.90 | 1.15 (1.10 - 1.20) |  |
| rs780094 | 2p23.3 | *GCKR* | G | 0.44 | 1.11 (1.01 - 1.22) |  |
| rs780094 | 2p23.3 | *GCKR* | G | 0.65 | 1.09 (1.02 - 1.16) |  |
| rs3792267 | 2q37.3 | *CAPN10* | G | 0.23 | 1.19 (1.07 - 1.33) |  |
| rs4607103 | 3p14.1 | *ADAMTS9* | C | 0.76 | 1.09 (1.06 - 1.12) |  |
| rs1801282 | 3p25.2 | *PPARG* | C | 0.84 | 1.28 (0.95 - 1.69) |  |
| rs1801282 | 3p25.2 | *PPARG* | C | 0.86 | 1.14 (1.08 - 1.20) |  |
| rs17036101 | 3p25.2 | *SYN2 - GSTM5P1* | G | 0.93 | 1.15 (1.10 - 1.21) |  |
| rs1470579 | 3q27.2 | *IGF2BP2* | C | 0.33 | 1.18 (1.07 - 1.31) |  |
| rs4402960 | 3q27.2 | *IGF2BP2* | T | 0.29* | 1.14 (1.11 - 1.17) |  |
| rs4402960 | 3q27.2 | *IGF2BP2* | T | 0.29 | 1.14 (1.11 - 1.18) |  |
| rs4402960 | 3q27.2 | *IGF2BP2* | T | 0.29* | 1.17 (1.10 - 1.25) |  |
| rs10010131 | 4p16.1 | *WFS1* | G | 0.63 | 1.11 (1.08 - 1.16) |  |
| rs10010131 | 4p16.1 | *WFS1* | G | 0.63* | 1.12 (1.09 - 1.16) |  |
| rs1801214 | 4p16.1 | *WFS1* | T | 0.63* | 1.13 (1.08 - 1.18) |  |
| rs9472138 | 6p21.1 | *VEGFA* | T | 0.28 | 1.06 (1.04 - 1.09) |  |
| rs10946398 | 6p22.3 | *CDKAL1* | C | 0.32* | 1.12 (1.08 - 1.16) |  |
| rs7754840 | 6p22.3 | *CDKAL1* | C | 0.41 | 1.28 (1.17 - 1.41) |  |
| rs7754840 | 6p22.3 | *CDKAL1* | C | 0.31 | 1.12 (1.08 - 1.16) |  |
| rs7756992 | 6p22.3 | *CDKAL1* | G | 0.27* | 1.16 (1.13 - 1.19) |  |
| rs7756992 | 6p22.3 | *CDKAL1* | G | 0.26 | 1.20 (1.13 - 1.27) |  |
| rs1799884 | 7p13 | *GCK* | A | 0.15 | 1.28 (1.06 - 1.53) |  |
| rs864745 | 7p15.1 | *JAZF1* | T | 0.50 | 1.10 (1.07 - 1.13) |  |
| rs1800795 | 7p15.3 | *IL6* | G | 0.82* | 1.10 (1.01 - 1.20) |  |
| rs13266634 | 8q24.11 | *SLC30A8* | C | 0.71* | 1.16 (1.13 - 1.19) |  |
| rs13266634 | 8q24.11 | *SLC30A8* | C | 0.65 | 1.12 (1.07 - 1.16) |  |
| rs3802177 | 8q24.11 | *SLC30A8* | G | 0.56 | 1.16 (1.05 - 1.27) |  |
| rs10811161 | 9p21.3 | *CDKN2B-AS1 - DMRTA1* | T | 0.84* | 1.22 (1.18 - 1.26) |  |
| rs10811661 | 9p21.3 | *CDKN2B-AS1 - DMRTA1* | T | 0.55 | 1.27 (1.15 - 1.40) |  |
| rs10811661 | 9p21.3 | *CDKN2B-AS1 - DMRTA1* | T | 0.83 | 1.20 (1.14 - 1.25) |  |
| rs10811661 | 9p21.3 | *CDKN2B-AS1 - DMRTA1* | T | 0.84* | 1.20 (1.14 - 1.25) |  |
| rs564398 | 9p21.3 | *CDKN2B-AS1 - DMRTA1* | T | 0.59* | 1.12 (1.07 - 1.17) |  |
| rs12779790 | 10p13 | *CDC123 - MIR4480* | G | 0.18 | 1.11 (1.07 - 1.14) |  |
| rs1111875 | 10q23.33 | *HHEX* | C | 0.53 | 1.13 (1.08 - 1.17) |  |
| rs5015480 | 10q23.33 | *HHEX* | C | 0.55* | 1.17 (1.11 - 1.24) |  |
| rs7923837 | 10q23.33 | *HHEX* | G | 0.19 | 1.27 (1.13 - 1.43) |  |
| rs7923837 | 10q23.33 | *HHEX* | G | 0.61* | 1.23 (1.18 - 1.29) |  |
| rs7901695 | 10q25.2 | *TCF7L2* | C | 0.32* | 1.37 (1.31 - 1.43) |  |
| rs7903146 | 10q25.2 | *TCF7L2* | T | 0.31* | 1.46 (1.42 - 1.51) |  |
| rs7903146 | 10q25.2 | *TCF7L2* | T | 0.31* | 1.54 (1.39 - 1.70) |  |
| rs7903146 | 10q25.2 | *TCF7L2* | T | 0.30 | 1.47 (1.33 - 1.62) |  |
| rs7903146 | 10q25.2 | *TCF7L2* | T | 0.04 | 1.48 (1.20 - 1.84) |  |
| rs7903146 | 10q25.2 | *TCF7L2* | T | 0.26 | 1.37 (1.31 - 1.43) |  |
| rs7903146 | 10q25.2 | *TCF7L2* | T | 0.31* | 1.37 (1.28 - 1.47) |  |
| rs11037909 | 11p11.2 | *EXT2* | T | 0.74 | 1.18 (1.08 - 1.29) |  |
| rs7480010 | 11p12 | *LOC387761* | G | 0.30 | 1.17 (1.08 - 1.27) |  |
| rs5215 | 11p15.1 | *KCNJ11* | C | 0.34* | 1.16 (1.09 - 1.23) |  |
| rs5219 | 11p15.1 | *KCNJ11* | T | 0.36 | 1.18 (1.04 - 1.34) |  |
| rs5219 | 11p15.1 | *KCNJ11* | T | 0.47 | 1.14 (1.10 - 1.19) |  |
| rs2237892 | 11p15.4 | *KCNQ1* | C | 0.65 | 1.53 (1.38 - 1.70) |  |
| rs2237892 | 11p15.4 | *KCNQ1* | C | 0.61 | 1.42 (1.34 - 1.49) |  |
| rs2237895 | 11p15.4 | *KCNQ1* | C | 0.40 | 1.19 (1.14 - 1.25) |  |
| rs2283228 | 11p15.4 | *KCNQ1* | A | 0.58 | 1.26 (1.18 - 1.34) |  |
| rs689 | 11p15.5 | *INS* | A | 0.28 | 1.16 (0.95 - 1.41) |  |
| rs10830963 | 11q14.3 | *MTNR1B* | G | 0.28 | 1.09 (1.05 - 1.12) |  |
| rs1387153 | 11q14.3 | *MTNR1B* | T | 0.28 | 1.09 (1.06 - 1.11) |  |
| rs1153188 | 12q13.2 | *DCD* | A | 0.73 | 1.08 (1.05 - 1.11) |  |
| rs7961581 | 12q21.1 | *TSPAN8 - LGR5* | C | 0.27 | 1.09 (1.06 - 1.12) |  |
| rs1800574 | 12q24.31 | *HNF1A* | T | 0.03 | 1.31 (1.08 - 1.57) |  |
| rs8050136 | 16q12.2 | *FTO* | A | 0.42* | 1.11 (1.08 - 1.15) |  |
| rs8050136 | 16q12.2 | *FTO* | A | 0.38 | 1.17 (1.12 - 1.22) |  |
| rs8050136 | 16q12.2 | *FTO* | A | 0.38 | 1.17 (1.12 - 1.22) |  |
| rs8050136 | 16q12.2 | *FTO* | A | 0.42* | 1.17 (1.12 - 1.22) |  |
| rs8050136 | 16q12.2 | *FTO* | A | 0.42* | 1.15 (1.09 - 1.22) |  |
| rs4430796 | 17q12 | *HNF1B* | A | 0.49 | 1.22 (1.15 - 1.30) |  |
| rs4430796 | 17q12 | *HNF1B* | G | 0.47* | 1.14 (1.08 - 1.20) |  |
| rs4430796 | 17q12 | *HNF1B* | G | 0.28 | 1.16 (1.05 - 1.29) |  |
| rs7501939 | 17q12 | *HNF1B* | C | 0.58 | 1.19 (1.12 - 1.26) |  |
| rs757210 | 17q12 | *HNF1B* | A | 0.43 | 1.12 (1.07 - 1.18) |  |
| rs1056137 | 20p12.3 | *KCNJ11* | A | 0.36 | 1.18 (1.04 - 1.34) |  |

**Supplementary Table 2** Risk allele frequencies of 18 single nucleotide polymorphisms from two prediction studies on type 2 diabetes and their corresponding values in the cited genome-wide association studies

| **Gene** | **SNP** | **Frequency in prediction study** | |  | **Frequency in cited GWAS*** | |
| --- | --- | --- | --- | --- | --- | --- |
|  |  | **GoDARTS study** | **Rotterdam study** |  | **GoDARTS study** | **Rotterdam study** |
| *ADAM30/*  *NOTCH2* | rs2641348† | 0.11 | 0.12 |  | 0.11 | |
| *ADAMTS9* | rs4607103‡‡ | 0.77 | 0.76 |  | 0.76 | |
| *CDC123* | rs12779790¥ | 0.20 | 0.18 |  | 0.18 | |
| *CDKAL1* | rs10946398§ | 0.34 | 0.31 |  | 0.31^1^ | |
| *CDKN2A/2B* | rs10811661 | 0.85 | 0.81 |  | 0.83 | |
| *CDKN2A/2B* | rs564398‡ | 0.59 | 0.57 |  | 0.59 | |
| *FTO* | rs8050136 | 0.41 | 0.37 |  | 0.42 | |
| *HHEX-IDE* | rs1111875 | 0.62 | 0.59 |  | 0.53 | |
| *IGF2BP2* | rs4402960 | 0.33 | 0.30 |  | 0.29 | |
| *JAZF1* | rs864745§§ | 0.50 | 0.52 |  | 0.50 | |
| *KCNJ11* | rs5219 | 0.36 | 0.37 |  | 0.36 | 0.47 |
| *PPARG* | rs1801282 | 0.87 | 0.88 |  | 0.86 | |
| *SLC30A8* | rs13266634 | 0.70 | 0.70 |  | 0.65 | |
| *TCF2* | rs757210†† | 0.37 | 0.49 |  | 0.43 | 0.49^1^ |
| *TCF7L2* | rs7903146 | 0.30 | 0.29 |  | 0.30 | 0.31 |
| *THADA* | rs7578597 | 0.91 | 0.88 |  | 0.90 | |
| *TSPAN8/*  *LGR5* | rs7961581¶ | 0.29 | 0.29 |  | 0.27 | |
| *WFS1* | rs10010131** | 0.60 | 0.60 |  | 0.63 | |

Table is adapted from . The risk models of the GoDARTS study and the Rotterdam Study included the same 18 genes and both had an AUC of 0.60. The AUC values from simulated data were the same and both were 0.61. The SNPs listed in the table are those used by the GoDARTS study. For several genes, the Rotterdam Study used different SNPs that were in linkage disequilibrium: †rs1493694, r^2^ = 0.74; ‡rs1412829, r^2^ = 0.97; §rs7754840, r^2^ = 1.00; ¶rs1353362, r^2^ = 0.96; ¥rs11257622; r^2^ = 0.83; **rs10012946, r^2^ = 1.00; ††rs4430796, r^2^ = 0.61; ‡‡rs4411878, r^2^ = 0.95; §§rs1635852, r^2^ = 0.97.

SNP, single nucleotide polymorphism; GWAS, genome-wide association study.

*When only one value is presented, both prediction studies cited the same GWAS.

^1^ GWAS studies reported for the SNP used by the Rotterdam study; all others are for SNPs used by the GoDARTS study.

**Supplementary Figure 1** Quintile plots presenting the ORs with 95% CIs for quintiles of genetic risk scores in 10 iterations

A. Considering the same sample size and risk thresholds from the prediction study. Note that the sizes of the groups are not exactly the same when the risk thresholds are the same between iterations.


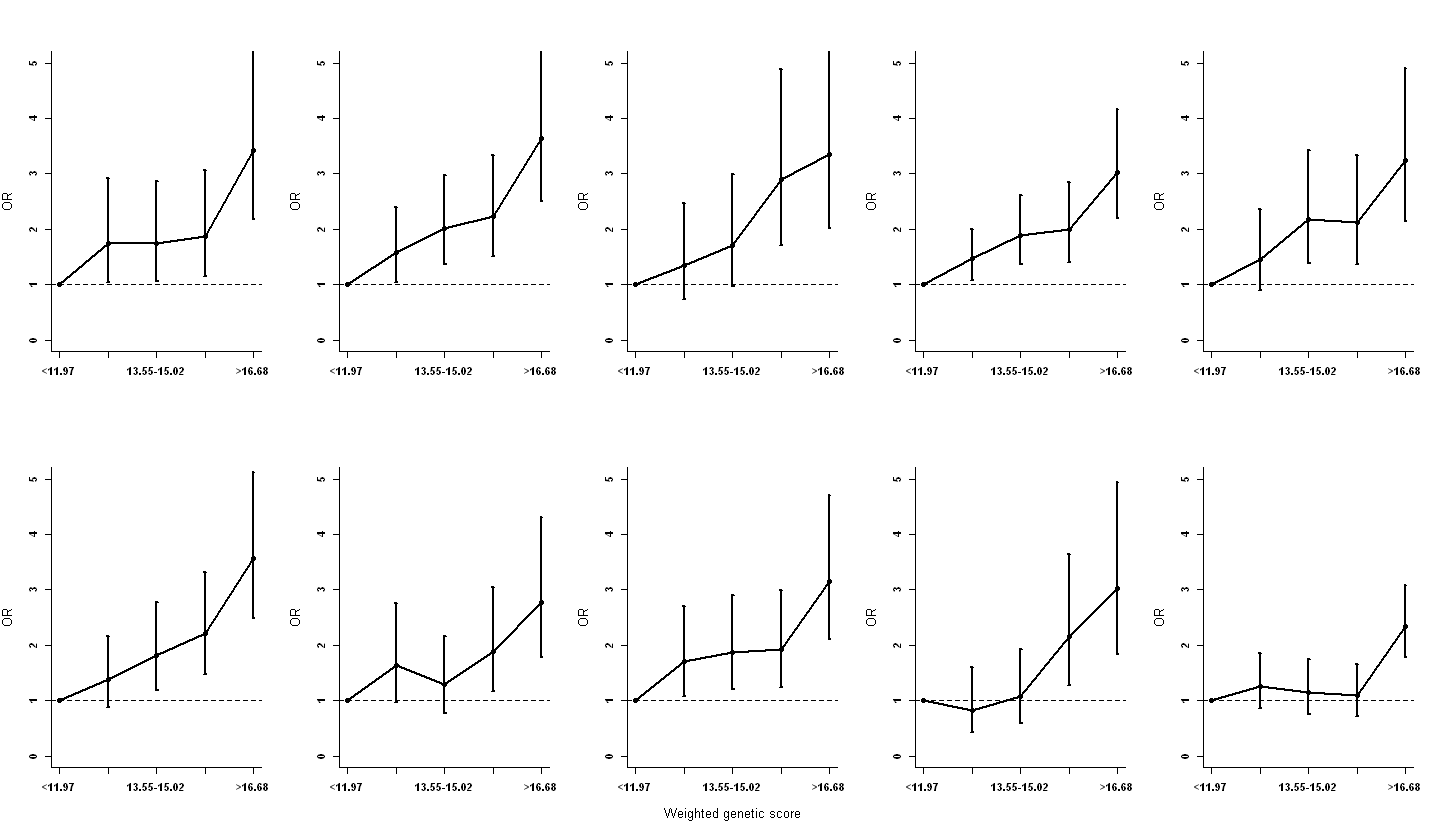


B. Considering the same sample size as reported in the prediction study but risk thresholds from the simulation study. Note that the risk thresholds might differ between iterations to split the population in quintiles.


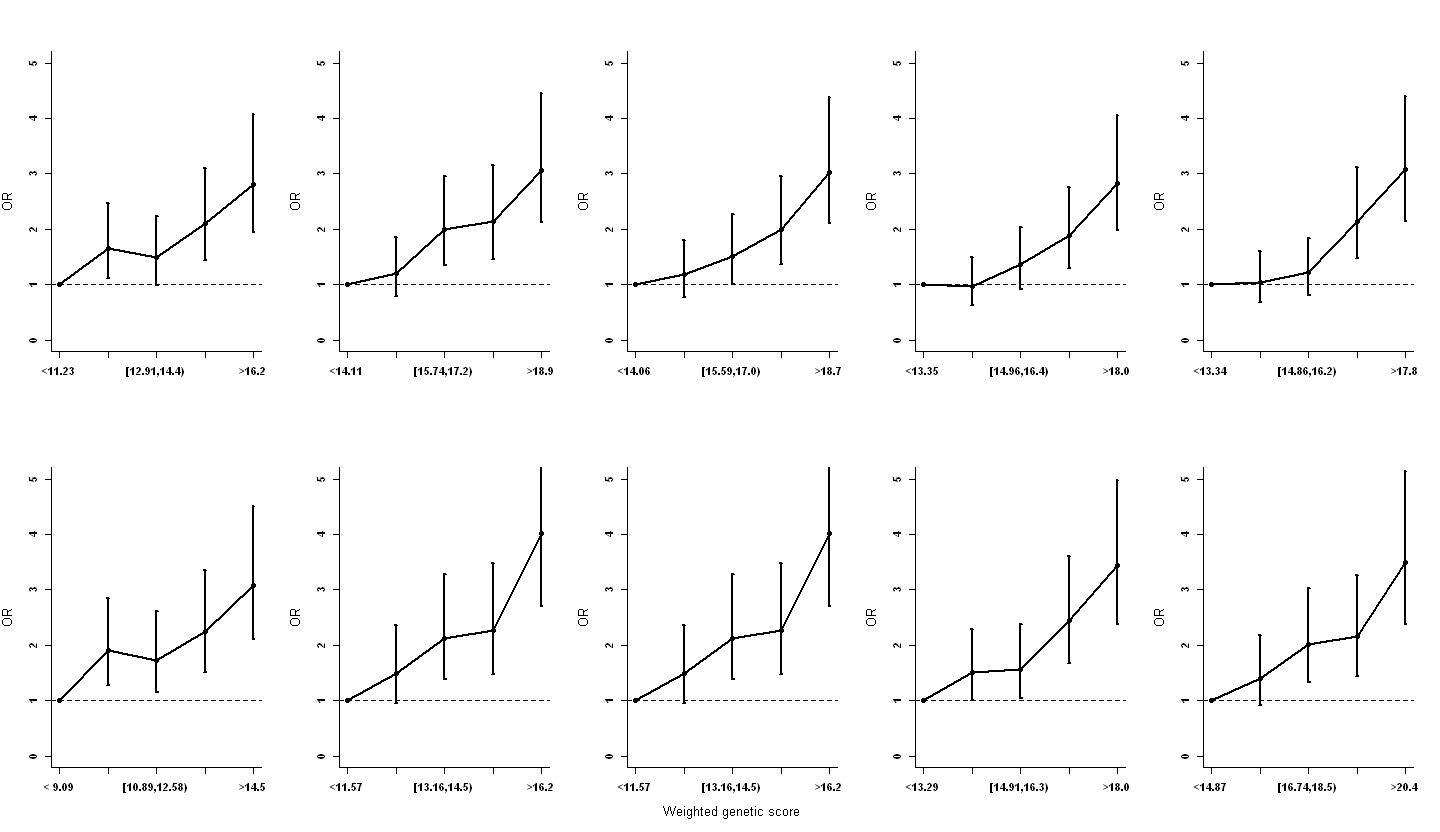


C. Considering the risk threshold from the prediction study and a sample size of 100,000. Note that the sizes of the groups are not exactly the same because the thresholds are taken from the prediction study.


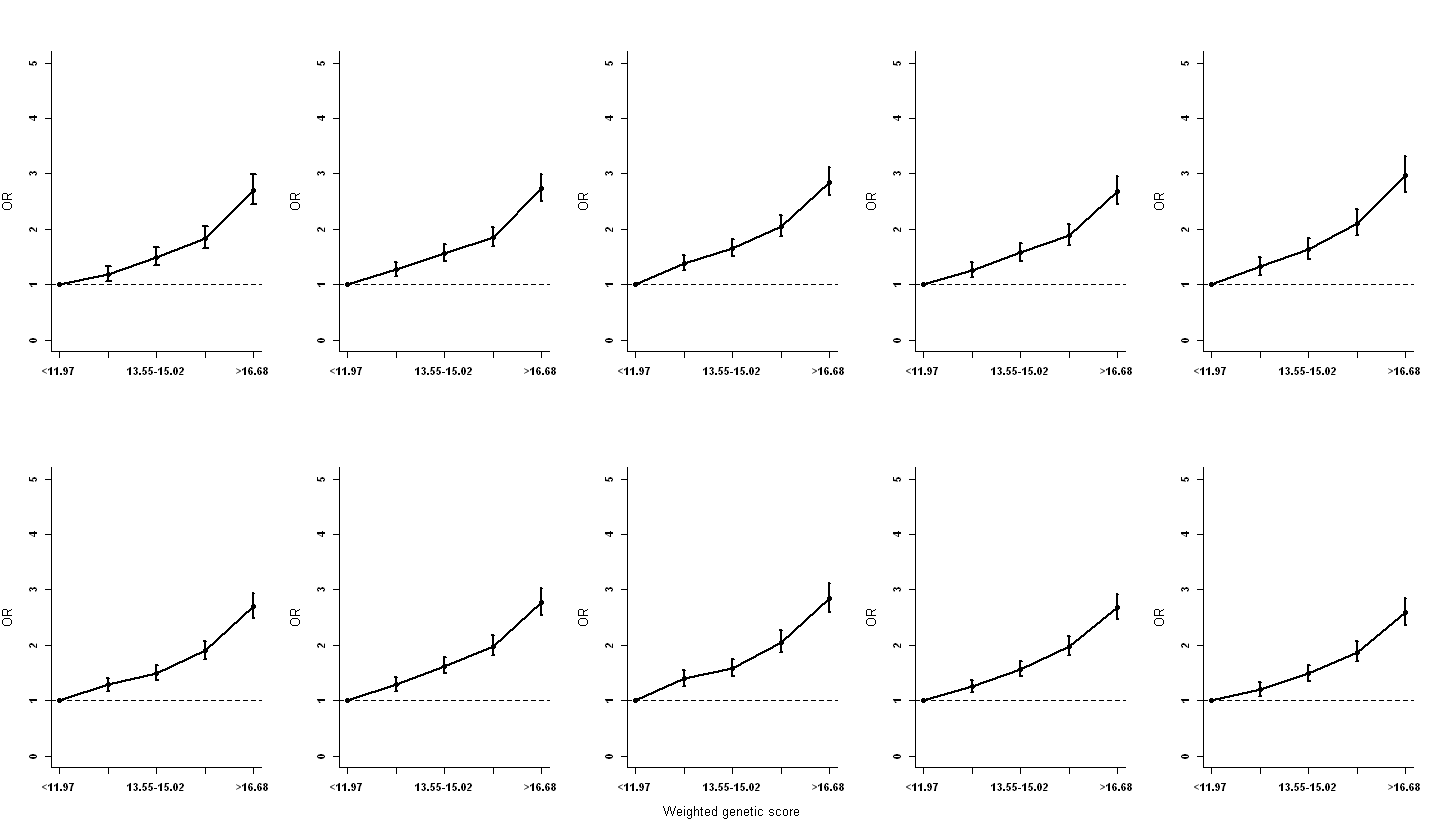


**References**

1000 Genomes Project Consortium (2012). An integrated map of genetic variation from 1,092 human genomes. *Nature* 491**,** 56-65. doi: 10.1038/nature11632.

Al Olama, A.A., Kote-Jarai, Z., Giles, G.G., Guy, M., Morrison, J., Severi, G., Leongamornlert, D.A., Tymrakiewicz, M., Jhavar, S., Saunders, E., Hopper, J.L., Southey, M.C., Muir, K.R., English, D.R., Dearnaley, D.P., Ardern-Jones, A.T., Hall, A.L., O'brien, L.T., Wilkinson, R.A., Sawyer, E., Lophatananon, A., Oncology, U.K.G.P.C.S.C.B.a.O.U.S.S.O., Cancer, U.K.P.T.F., Treatment Study, C., Horwich, A., Huddart, R.A., Khoo, V.S., Parker, C.C., Woodhouse, C.J., Thompson, A., Christmas, T., Ogden, C., Cooper, C., Donovan, J.L., Hamdy, F.C., Neal, D.E., Eeles, R.A., and Easton, D.F. (2009). Multiple loci on 8q24 associated with prostate cancer susceptibility. *Nat Genet* 41**,** 1058-1060.

Altshuler, D., Hirschhorn, J.N., Klannemark, M., Lindgren, C.M., Vohl, M.C., Nemesh, J., Lane, C.R., Schaffner, S.F., Bolk, S., Brewer, C., Tuomi, T., Gaudet, D., Hudson, T.J., Daly, M., Groop, L., and Lander, E.S. (2000). The common PPARgamma Pro12Ala polymorphism is associated with decreased risk of type 2 diabetes. *Nat Genet* 26**,** 76-80. doi: 10.1038/79216 [doi].

Awata, T., Kawasaki, E., Tanaka, S., Ikegami, H., Maruyama, T., Shimada, A., Nakanishi, K., Kobayashi, T., Iizuka, H., Uga, M., Kawabata, Y., Kanazawa, Y., Kurihara, S., Osaki, M., Katayama, S., and Japanese Study Group on Type 1 Diabetes, G. (2009). Association of type 1 diabetes with two Loci on 12q13 and 16p13 and the influence coexisting thyroid autoimmunity in Japanese. *J Clin Endocrinol Metab* 94**,** 231-235.

Broderick, P., Carvajal-Carmona, L., Pittman, A.M., Webb, E., Howarth, K., Rowan, A., Lubbe, S., Spain, S., Sullivan, K., Fielding, S., Jaeger, E., Vijayakrishnan, J., Kemp, Z., Gorman, M., Chandler, I., Papaemmanuil, E., Penegar, S., Wood, W., Sellick, G., Qureshi, M., Teixeira, A., Domingo, E., Barclay, E., Martin, L., Sieber, O., Consortium, C., Kerr, D., Gray, R., Peto, J., Cazier, J.B., Tomlinson, I., and Houlston, R.S. (2007). A genome-wide association study shows that common alleles of SMAD7 influence colorectal cancer risk. *Nat Genet* 39**,** 1315-1317.

Cauchi, S., El Achhab, Y., Choquet, H., Dina, C., Krempler, F., Weitgasser, R., Nejjari, C., Patsch, W., Chikri, M., Meyre, D., and Froguel, P. (2007). TCF7L2 is reproducibly associated with type 2 diabetes in various ethnic groups: a global meta-analysis. *J Mol Med (Berl)* 85**,** 777-782. doi: 10.1007/s00109-007-0203-4 [doi].

Chen, W., Stambolian, D., Edwards, A.O., Branham, K.E., Othman, M., Jakobsdottir, J., Tosakulwong, N., Pericak-Vance, M.A., Campochiaro, P.A., Klein, M.L., Tan, P.L., Conley, Y.P., Kanda, A., Kopplin, L., Li, Y., Augustaitis, K.J., Karoukis, A.J., Scott, W.K., Agarwal, A., Kovach, J.L., Schwartz, S.G., Postel, E.A., Brooks, M., Baratz, K.H., Brown, W.L., Complications of Age-Related Macular Degeneration Prevention Trial Research, G., Brucker, A.J., Orlin, A., Brown, G., Ho, A., Regillo, C., Donoso, L., Tian, L., Kaderli, B., Hadley, D., Hagstrom, S.A., Peachey, N.S., Klein, R., Klein, B.E., Gotoh, N., Yamashiro, K., Ferris Iii, F., Fagerness, J.A., Reynolds, R., Farrer, L.A., Kim, I.K., Miller, J.W., Corton, M., Carracedo, A., Sanchez-Salorio, M., Pugh, E.W., Doheny, K.F., Brion, M., Deangelis, M.M., Weeks, D.E., Zack, D.J., Chew, E.Y., Heckenlively, J.R., Yoshimura, N., Iyengar, S.K., Francis, P.J., Katsanis, N., Seddon, J.M., Haines, J.L., Gorin, M.B., Abecasis, G.R., and Swaroop, A. (2010). Genetic variants near TIMP3 and high-density lipoprotein-associated loci influence susceptibility to age-related macular degeneration. *Proc Natl Acad Sci U S A* 107**,** 7401-7406.

Cipriani, V., Leung, H.T., Plagnol, V., Bunce, C., Khan, J.C., Shahid, H., Moore, A.T., Harding, S.P., Bishop, P.N., Hayward, C., Campbell, S., Armbrecht, A.M., Dhillon, B., Deary, I.J., Campbell, H., Dunlop, M., Dominiczak, A.F., Mann, S.S., Jenkins, S.A., Webster, A.R., Bird, A.C., Lathrop, M., Zelenika, D., Souied, E.H., Sahel, J.A., Leveillard, T., French, A.M.D.I., Cree, A.J., Gibson, J., Ennis, S., Lotery, A.J., Wright, A.F., Clayton, D.G., and Yates, J.R. (2012). Genome-wide association study of age-related macular degeneration identifies associated variants in the TNXB-FKBPL-NOTCH4 region of chromosome 6p21.3. *Hum Mol Genet* 21**,** 4138-4150.

Duerr, R.H., Taylor, K.D., Brant, S.R., Rioux, J.D., Silverberg, M.S., Daly, M.J., Steinhart, A.H., Abraham, C., Regueiro, M., Griffiths, A., Dassopoulos, T., Bitton, A., Yang, H., Targan, S., Datta, L.W., Kistner, E.O., Schumm, L.P., Lee, A.T., Gregersen, P.K., Barmada, M.M., Rotter, J.I., Nicolae, D.L., and Cho, J.H. (2006). A genome-wide association study identifies IL23R as an inflammatory bowel disease gene. *Science* 314**,** 1461-1463.

Duggan, D., Zheng, S.L., Knowlton, M., Benitez, D., Dimitrov, L., Wiklund, F., Robbins, C., Isaacs, S.D., Cheng, Y., Li, G., Sun, J., Chang, B.L., Marovich, L., Wiley, K.E., Balter, K., Stattin, P., Adami, H.O., Gielzak, M., Yan, G., Sauvageot, J., Liu, W., Kim, J.W., Bleecker, E.R., Meyers, D.A., Trock, B.J., Partin, A.W., Walsh, P.C., Isaacs, W.B., Gronberg, H., Xu, J., and Carpten, J.D. (2007). Two genome-wide association studies of aggressive prostate cancer implicate putative prostate tumor suppressor gene DAB2IP. *J Natl Cancer Inst* 99**,** 1836-1844.

Economou, M., Trikalinos, T.A., Loizou, K.T., Tsianos, E.V., and Ioannidis, J.P. (2004). Differential effects of NOD2 variants on Crohn's disease risk and phenotype in diverse populations: a metaanalysis. *Am J Gastroenterol* 99**,** 2393-2404.

Eeles, R.A., Kote-Jarai, Z., Al Olama, A.A., Giles, G.G., Guy, M., Severi, G., Muir, K., Hopper, J.L., Henderson, B.E., Haiman, C.A., Schleutker, J., Hamdy, F.C., Neal, D.E., Donovan, J.L., Stanford, J.L., Ostrander, E.A., Ingles, S.A., John, E.M., Thibodeau, S.N., Schaid, D., Park, J.Y., Spurdle, A., Clements, J., Dickinson, J.L., Maier, C., Vogel, W., Dork, T., Rebbeck, T.R., Cooney, K.A., Cannon-Albright, L., Chappuis, P.O., Hutter, P., Zeegers, M., Kaneva, R., Zhang, H.W., Lu, Y.J., Foulkes, W.D., English, D.R., Leongamornlert, D.A., Tymrakiewicz, M., Morrison, J., Ardern-Jones, A.T., Hall, A.L., O'brien, L.T., Wilkinson, R.A., Saunders, E.J., Page, E.C., Sawyer, E.J., Edwards, S.M., Dearnaley, D.P., Horwich, A., Huddart, R.A., Khoo, V.S., Parker, C.C., Van As, N., Woodhouse, C.J., Thompson, A., Christmas, T., Ogden, C., Cooper, C.S., Southey, M.C., Lophatananon, A., Liu, J.F., Kolonel, L.N., Le Marchand, L., Wahlfors, T., Tammela, T.L., Auvinen, A., Lewis, S.J., Cox, A., Fitzgerald, L.M., Koopmeiners, J.S., Karyadi, D.M., Kwon, E.M., Stern, M.C., Corral, R., Joshi, A.D., Shahabi, A., Mcdonnell, S.K., Sellers, T.A., Pow-Sang, J., Chambers, S., Aitken, J., Gardiner, R.A., Batra, J., Kedda, M.A., Lose, F., Polanowski, A., Patterson, B., Serth, J., Meyer, A., Luedeke, M., Stefflova, K., Ray, A.M., Lange, E.M., Farnham, J., Khan, H., Slavov, C., Mitkova, A., Cao, G., et al. (2009). Identification of seven new prostate cancer susceptibility loci through a genome-wide association study. *Nat Genet* 41**,** 1116-1121.

Eeles, R.A., Kote-Jarai, Z., Giles, G.G., Olama, A.A., Guy, M., Jugurnauth, S.K., Mulholland, S., Leongamornlert, D.A., Edwards, S.M., Morrison, J., Field, H.I., Southey, M.C., Severi, G., Donovan, J.L., Hamdy, F.C., Dearnaley, D.P., Muir, K.R., Smith, C., Bagnato, M., Ardern-Jones, A.T., Hall, A.L., O'brien, L.T., Gehr-Swain, B.N., Wilkinson, R.A., Cox, A., Lewis, S., Brown, P.M., Jhavar, S.G., Tymrakiewicz, M., Lophatananon, A., Bryant, S.L., Collaborators, U.K.G.P.C.S., British Association of Urological Surgeons' Section Of, O., Collaborators, U.K.P.S., Horwich, A., Huddart, R.A., Khoo, V.S., Parker, C.C., Woodhouse, C.J., Thompson, A., Christmas, T., Ogden, C., Fisher, C., Jamieson, C., Cooper, C.S., English, D.R., Hopper, J.L., Neal, D.E., and Easton, D.F. (2008). Multiple newly identified loci associated with prostate cancer susceptibility. *Nat Genet* 40**,** 316-321.

Flicek, P., Ahmed, I., Amode, M.R., Barrell, D., Beal, K., Brent, S., Carvalho-Silva, D., Clapham, P., Coates, G., Fairley, S., Fitzgerald, S., Gil, L., Garcia-Giron, C., Gordon, L., Hourlier, T., Hunt, S., Juettemann, T., Kahari, A.K., Keenan, S., Komorowska, M., Kulesha, E., Longden, I., Maurel, T., Mclaren, W.M., Muffato, M., Nag, R., Overduin, B., Pignatelli, M., Pritchard, B., Pritchard, E., Riat, H.S., Ritchie, G.R., Ruffier, M., Schuster, M., Sheppard, D., Sobral, D., Taylor, K., Thormann, A., Trevanion, S., White, S., Wilder, S.P., Aken, B.L., Birney, E., Cunningham, F., Dunham, I., Harrow, J., Herrero, J., Hubbard, T.J., Johnson, N., Kinsella, R., Parker, A., Spudich, G., Yates, A., Zadissa, A., and Searle, S.M. (2013). Ensembl 2013. *Nucleic Acids Res* 41**,** D48-55.

Fritsche, L.G., Freitag-Wolf, S., Bettecken, T., Meitinger, T., Keilhauer, C.N., Krawczak, M., and Weber, B.H. (2009). Age-related macular degeneration and functional promoter and coding variants of the apolipoprotein E gene. *Hum Mutat* 30**,** 1048-1053.

Gloyn, A.L., Weedon, M.N., Owen, K.R., Turner, M.J., Knight, B.A., Hitman, G., Walker, M., Levy, J.C., Sampson, M., Halford, S., Mccarthy, M.I., Hattersley, A.T., and Frayling, T.M. (2003). Large-scale association studies of variants in genes encoding the pancreatic beta-cell KATP channel subunits Kir6.2 (KCNJ11) and SUR1 (ABCC8) confirm that the KCNJ11 E23K variant is associated with type 2 diabetes. *Diabetes* 52**,** 568-572.

Gold, B., Merriam, J.E., Zernant, J., Hancox, L.S., Taiber, A.J., Gehrs, K., Cramer, K., Neel, J., Bergeron, J., Barile, G.R., Smith, R.T., Group, A.M.D.G.C.S., Hageman, G.S., Dean, M., and Allikmets, R. (2006). Variation in factor B (BF) and complement component 2 (C2) genes is associated with age-related macular degeneration. *Nat Genet* 38**,** 458-462.

Grant, S.F., Thorleifsson, G., Reynisdottir, I., Benediktsson, R., Manolescu, A., Sainz, J., Helgason, A., Stefansson, H., Emilsson, V., Helgadottir, A., Styrkarsdottir, U., Magnusson, K.P., Walters, G.B., Palsdottir, E., Jonsdottir, T., Gudmundsdottir, T., Gylfason, A., Saemundsdottir, J., Wilensky, R.L., Reilly, M.P., Rader, D.J., Bagger, Y., Christiansen, C., Gudnason, V., Sigurdsson, G., Thorsteinsdottir, U., Gulcher, J.R., Kong, A., and Stefansson, K. (2006). Variant of transcription factor 7-like 2 (TCF7L2) gene confers risk of type 2 diabetes. *Nat Genet* 38**,** 320-323. doi: ng1732 [pii]

10.1038/ng1732 [doi].

Gudmundsson, J., Sulem, P., Gudbjartsson, D.F., Blondal, T., Gylfason, A., Agnarsson, B.A., Benediktsdottir, K.R., Magnusdottir, D.N., Orlygsdottir, G., Jakobsdottir, M., Stacey, S.N., Sigurdsson, A., Wahlfors, T., Tammela, T., Breyer, J.P., Mcreynolds, K.M., Bradley, K.M., Saez, B., Godino, J., Navarrete, S., Fuertes, F., Murillo, L., Polo, E., Aben, K.K., Van Oort, I.M., Suarez, B.K., Helfand, B.T., Kan, D., Zanon, C., Frigge, M.L., Kristjansson, K., Gulcher, J.R., Einarsson, G.V., Jonsson, E., Catalona, W.J., Mayordomo, J.I., Kiemeney, L.A., Smith, J.R., Schleutker, J., Barkardottir, R.B., Kong, A., Thorsteinsdottir, U., Rafnar, T., and Stefansson, K. (2009). Genome-wide association and replication studies identify four variants associated with prostate cancer susceptibility. *Nat Genet* 41**,** 1122-1126.

Gudmundsson, J., Sulem, P., Manolescu, A., Amundadottir, L.T., Gudbjartsson, D., Helgason, A., Rafnar, T., Bergthorsson, J.T., Agnarsson, B.A., Baker, A., Sigurdsson, A., Benediktsdottir, K.R., Jakobsdottir, M., Xu, J., Blondal, T., Kostic, J., Sun, J., Ghosh, S., Stacey, S.N., Mouy, M., Saemundsdottir, J., Backman, V.M., Kristjansson, K., Tres, A., Partin, A.W., Albers-Akkers, M.T., Godino-Ivan Marcos, J., Walsh, P.C., Swinkels, D.W., Navarrete, S., Isaacs, S.D., Aben, K.K., Graif, T., Cashy, J., Ruiz-Echarri, M., Wiley, K.E., Suarez, B.K., Witjes, J.A., Frigge, M., Ober, C., Jonsson, E., Einarsson, G.V., Mayordomo, J.I., Kiemeney, L.A., Isaacs, W.B., Catalona, W.J., Barkardottir, R.B., Gulcher, J.R., Thorsteinsdottir, U., Kong, A., and Stefansson, K. (2007a). Genome-wide association study identifies a second prostate cancer susceptibility variant at 8q24. *Nat Genet* 39**,** 631-637.

Gudmundsson, J., Sulem, P., Rafnar, T., Bergthorsson, J.T., Manolescu, A., Gudbjartsson, D., Agnarsson, B.A., Sigurdsson, A., Benediktsdottir, K.R., Blondal, T., Jakobsdottir, M., Stacey, S.N., Kostic, J., Kristinsson, K.T., Birgisdottir, B., Ghosh, S., Magnusdottir, D.N., Thorlacius, S., Thorleifsson, G., Zheng, S.L., Sun, J., Chang, B.L., Elmore, J.B., Breyer, J.P., Mcreynolds, K.M., Bradley, K.M., Yaspan, B.L., Wiklund, F., Stattin, P., Lindstrom, S., Adami, H.O., Mcdonnell, S.K., Schaid, D.J., Cunningham, J.M., Wang, L., Cerhan, J.R., St Sauver, J.L., Isaacs, S.D., Wiley, K.E., Partin, A.W., Walsh, P.C., Polo, S., Ruiz-Echarri, M., Navarrete, S., Fuertes, F., Saez, B., Godino, J., Weijerman, P.C., Swinkels, D.W., Aben, K.K., Witjes, J.A., Suarez, B.K., Helfand, B.T., Frigge, M.L., Kristjansson, K., Ober, C., Jonsson, E., Einarsson, G.V., Xu, J., Gronberg, H., Smith, J.R., Thibodeau, S.N., Isaacs, W.B., Catalona, W.J., Mayordomo, J.I., Kiemeney, L.A., Barkardottir, R.B., Gulcher, J.R., Thorsteinsdottir, U., Kong, A., and Stefansson, K. (2008). Common sequence variants on 2p15 and Xp11.22 confer susceptibility to prostate cancer. *Nat Genet* 40**,** 281-283.

Gudmundsson, J., Sulem, P., Steinthorsdottir, V., Bergthorsson, J.T., Thorleifsson, G., Manolescu, A., Rafnar, T., Gudbjartsson, D., Agnarsson, B.A., Baker, A., Sigurdsson, A., Benediktsdottir, K.R., Jakobsdottir, M., Blondal, T., Stacey, S.N., Helgason, A., Gunnarsdottir, S., Olafsdottir, A., Kristinsson, K.T., Birgisdottir, B., Ghosh, S., Thorlacius, S., Magnusdottir, D., Stefansdottir, G., Kristjansson, K., Bagger, Y., Wilensky, R.L., Reilly, M.P., Morris, A.D., Kimber, C.H., Adeyemo, A., Chen, Y., Zhou, J., So, W.Y., Tong, P.C., Ng, M.C., Hansen, T., Andersen, G., Borch-Johnsen, K., Jorgensen, T., Tres, A., Fuertes, F., Ruiz-Echarri, M., Asin, L., Saez, B., Van Boven, E., Klaver, S., Swinkels, D.W., Aben, K.K., Graif, T., Cashy, J., Suarez, B.K., Van Vierssen Trip, O., Frigge, M.L., Ober, C., Hofker, M.H., Wijmenga, C., Christiansen, C., Rader, D.J., Palmer, C.N., Rotimi, C., Chan, J.C., Pedersen, O., Sigurdsson, G., Benediktsson, R., Jonsson, E., Einarsson, G.V., Mayordomo, J.I., Catalona, W.J., Kiemeney, L.A., Barkardottir, R.B., Gulcher, J.R., Thorsteinsdottir, U., Kong, A., and Stefansson, K. (2007b). Two variants on chromosome 17 confer prostate cancer risk, and the one in TCF2 protects against type 2 diabetes. *Nat Genet* 39**,** 977-983.

Hageman, G.S., Anderson, D.H., Johnson, L.V., Hancox, L.S., Taiber, A.J., Hardisty, L.I., Hageman, J.L., Stockman, H.A., Borchardt, J.D., Gehrs, K.M., Smith, R.J., Silvestri, G., Russell, S.R., Klaver, C.C., Barbazetto, I., Chang, S., Yannuzzi, L.A., Barile, G.R., Merriam, J.C., Smith, R.T., Olsh, A.K., Bergeron, J., Zernant, J., Merriam, J.E., Gold, B., Dean, M., and Allikmets, R. (2005). A common haplotype in the complement regulatory gene factor H (HF1/CFH) predisposes individuals to age-related macular degeneration. *Proc Natl Acad Sci U S A* 102**,** 7227-7232.

Helgason, A., Palsson, S., Thorleifsson, G., Grant, S.F., Emilsson, V., Gunnarsdottir, S., Adeyemo, A., Chen, Y., Chen, G., Reynisdottir, I., Benediktsson, R., Hinney, A., Hansen, T., Andersen, G., Borch-Johnsen, K., Jorgensen, T., Schafer, H., Faruque, M., Doumatey, A., Zhou, J., Wilensky, R.L., Reilly, M.P., Rader, D.J., Bagger, Y., Christiansen, C., Sigurdsson, G., Hebebrand, J., Pedersen, O., Thorsteinsdottir, U., Gulcher, J.R., Kong, A., Rotimi, C., and Stefansson, K. (2007). Refining the impact of TCF7L2 gene variants on type 2 diabetes and adaptive evolution. *Nat Genet* 39**,** 218-225. doi: ng1960 [pii]

10.1038/ng1960 [doi].

Hindorff, L.A., Sethupathy, P., Junkins, H.A., Ramos, E.M., Mehta, J.P., Collins, F.S., and Manolio, T.A. (2009). Potential etiologic and functional implications of genome-wide association loci for human diseases and traits. *Proc Natl Acad Sci U S A* 106**,** 9362-9367.

Horikawa, Y., Miyake, K., Yasuda, K., Enya, M., Hirota, Y., Yamagata, K., Hinokio, Y., Oka, Y., Iwasaki, N., Iwamoto, Y., Yamada, Y., Seino, Y., Maegawa, H., Kashiwagi, A., Yamamoto, K., Tokunaga, K., Takeda, J., and Kasuga, M. (2008). Replication of genome-wide association studies of type 2 diabetes susceptibility in Japan. *J Clin Endocrinol Metab* 93**,** 3136-3141. doi: jc.2008-0452 [pii]

10.1210/jc.2008-0452 [doi].

Houlston, R.S., Cheadle, J., Dobbins, S.E., Tenesa, A., Jones, A.M., Howarth, K., Spain, S.L., Broderick, P., Domingo, E., Farrington, S., Prendergast, J.G., Pittman, A.M., Theodoratou, E., Smith, C.G., Olver, B., Walther, A., Barnetson, R.A., Churchman, M., Jaeger, E.E., Penegar, S., Barclay, E., Martin, L., Gorman, M., Mager, R., Johnstone, E., Midgley, R., Niittymaki, I., Tuupanen, S., Colley, J., Idziaszczyk, S., Consortium, C., Thomas, H.J., Lucassen, A.M., Evans, D.G., Maher, E.R., Consortium, C., Group, C.C., Group, C.C., Maughan, T., Dimas, A., Dermitzakis, E., Cazier, J.B., Aaltonen, L.A., Pharoah, P., Kerr, D.J., Carvajal-Carmona, L.G., Campbell, H., Dunlop, M.G., and Tomlinson, I.P. (2010). Meta-analysis of three genome-wide association studies identifies susceptibility loci for colorectal cancer at 1q41, 3q26.2, 12q13.13 and 20q13.33. *Nat Genet* 42**,** 973-977.

Houlston, R.S., Webb, E., Broderick, P., Pittman, A.M., Di Bernardo, M.C., Lubbe, S., Chandler, I., Vijayakrishnan, J., Sullivan, K., Penegar, S., Colorectal Cancer Association Study, C., Carvajal-Carmona, L., Howarth, K., Jaeger, E., Spain, S.L., Walther, A., Barclay, E., Martin, L., Gorman, M., Domingo, E., Teixeira, A.S., Co, R.G.I.C., Kerr, D., Cazier, J.B., Niittymaki, I., Tuupanen, S., Karhu, A., Aaltonen, L.A., Tomlinson, I.P., Farrington, S.M., Tenesa, A., Prendergast, J.G., Barnetson, R.A., Cetnarskyj, R., Porteous, M.E., Pharoah, P.D., Koessler, T., Hampe, J., Buch, S., Schafmayer, C., Tepel, J., Schreiber, S., Volzke, H., Chang-Claude, J., Hoffmeister, M., Brenner, H., Zanke, B.W., Montpetit, A., Hudson, T.J., Gallinger, S., International Colorectal Cancer Genetic Association, C., Campbell, H., and Dunlop, M.G. (2008). Meta-analysis of genome-wide association data identifies four new susceptibility loci for colorectal cancer. *Nat Genet* 40**,** 1426-1435.

Hu, C., Wang, C., Zhang, R., Ma, X., Wang, J., Lu, J., Qin, W., Bao, Y., Xiang, K., and Jia, W. (2009). Variations in KCNQ1 are associated with type 2 diabetes and beta cell function in a Chinese population. *Diabetologia* 52**,** 1322-1325. doi: 10.1007/s00125-009-1335-6 [doi].

Hughes, A.E., Orr, N., Esfandiary, H., Diaz-Torres, M., Goodship, T., and Chakravarthy, U. (2006). A common CFH haplotype, with deletion of CFHR1 and CFHR3, is associated with lower risk of age-related macular degeneration. *Nat Genet* 38**,** 1173-1177.

Huth, C., Heid, I.M., Vollmert, C., Gieger, C., Grallert, H., Wolford, J.K., Langer, B., Thorand, B., Klopp, N., Hamid, Y.H., Pedersen, O., Hansen, T., Lyssenko, V., Groop, L., Meisinger, C., Doring, A., Lowel, H., Lieb, W., Hengstenberg, C., Rathmann, W., Martin, S., Stephens, J.W., Ireland, H., Mather, H., Miller, G.J., Stringham, H.M., Boehnke, M., Tuomilehto, J., Boeing, H., Mohlig, M., Spranger, J., Pfeiffer, A., Wernstedt, I., Niklason, A., Lopez-Bermejo, A., Fernandez-Real, J.M., Hanson, R.L., Gallart, L., Vendrell, J., Tsiavou, A., Hatziagelaki, E., Humphries, S.E., Wichmann, H.E., Herder, C., and Illig, T. (2006). IL6 gene promoter polymorphisms and type 2 diabetes: joint analysis of individual participants' data from 21 studies. *Diabetes* 55**,** 2915-2921. doi: 55/10/2915 [pii]

10.2337/db06-0600 [doi].

Ikegami, H., Awata, T., Kawasaki, E., Kobayashi, T., Maruyama, T., Nakanishi, K., Shimada, A., Amemiya, S., Kawabata, Y., Kurihara, S., Tanaka, S., Kanazawa, Y., Mochizuki, M., and Ogihara, T. (2006). The association of CTLA4 polymorphism with type 1 diabetes is concentrated in patients complicated with autoimmune thyroid disease: a multicenter collaborative study in Japan. *J Clin Endocrinol Metab* 91**,** 1087-1092.

Jaeger, E., Webb, E., Howarth, K., Carvajal-Carmona, L., Rowan, A., Broderick, P., Walther, A., Spain, S., Pittman, A., Kemp, Z., Sullivan, K., Heinimann, K., Lubbe, S., Domingo, E., Barclay, E., Martin, L., Gorman, M., Chandler, I., Vijayakrishnan, J., Wood, W., Papaemmanuil, E., Penegar, S., Qureshi, M., Consortium, C., Farrington, S., Tenesa, A., Cazier, J.B., Kerr, D., Gray, R., Peto, J., Dunlop, M., Campbell, H., Thomas, H., Houlston, R., and Tomlinson, I. (2008). Common genetic variants at the CRAC1 (HMPS) locus on chromosome 15q13.3 influence colorectal cancer risk. *Nat Genet* 40**,** 26-28.

Janssens, A.C., and Van Duijn, C.M. (2009). Genome-based prediction of common diseases: methodological considerations for future research. *Genome Med* 1**,** 20. doi: 10.1186/gm20.

Johansson, M., Holmstrom, B., Hinchliffe, S.R., Bergh, A., Stenman, U.H., Hallmans, G., Wiklund, F., and Stattin, P. (2012). Combining 33 genetic variants with prostate-specific antigen for prediction of prostate cancer: longitudinal study. *Int J Cancer* 130**,** 129-137. doi: 10.1002/ijc.25986.

Kawasaki, E., Awata, T., Ikegami, H., Kobayashi, T., Maruyama, T., Nakanishi, K., Shimada, A., Uga, M., Kurihara, S., Kawabata, Y., Tanaka, S., Kanazawa, Y., Eguchi, K., and Japanese Study Group on Type 1 Diabetes, G. (2009). Genetic association between the interleukin-2 receptor-alpha gene and mode of onset of type 1 diabetes in the Japanese population. *J Clin Endocrinol Metab* 94**,** 947-952.

Kim, S.T., Cheng, Y., Hsu, F.C., Jin, T., Kader, A.K., Zheng, S.L., Isaacs, W.B., Xu, J., and Sun, J. (2010). Prostate cancer risk-associated variants reported from genome-wide association studies: meta-analysis and their contribution to genetic Variation. *Prostate* 70**,** 1729-1738.

Lango, H., Consortium, U.K.T.D.G., Palmer, C.N., Morris, A.D., Zeggini, E., Hattersley, A.T., Mccarthy, M.I., Frayling, T.M., and Weedon, M.N. (2008). Assessing the combined impact of 18 common genetic variants of modest effect sizes on type 2 diabetes risk. *Diabetes* 57**,** 3129-3135. doi: 10.2337/db08-0504.

Libioulle, C., Louis, E., Hansoul, S., Sandor, C., Farnir, F., Franchimont, D., Vermeire, S., Dewit, O., De Vos, M., Dixon, A., Demarche, B., Gut, I., Heath, S., Foglio, M., Liang, L., Laukens, D., Mni, M., Zelenika, D., Van Gossum, A., Rutgeerts, P., Belaiche, J., Lathrop, M., and Georges, M. (2007). Novel Crohn disease locus identified by genome-wide association maps to a gene desert on 5p13.1 and modulates expression of PTGER4. *PLoS Genet* 3**,** e58.

Lou, H., Yeager, M., Li, H., Bosquet, J.G., Hayes, R.B., Orr, N., Yu, K., Hutchinson, A., Jacobs, K.B., Kraft, P., Wacholder, S., Chatterjee, N., Feigelson, H.S., Thun, M.J., Diver, W.R., Albanes, D., Virtamo, J., Weinstein, S., Ma, J., Gaziano, J.M., Stampfer, M., Schumacher, F.R., Giovannucci, E., Cancel-Tassin, G., Cussenot, O., Valeri, A., Andriole, G.L., Crawford, E.D., Anderson, S.K., Tucker, M., Hoover, R.N., Fraumeni, J.F., Jr., Thomas, G., Hunter, D.J., Dean, M., and Chanock, S.J. (2009). Fine mapping and functional analysis of a common variant in MSMB on chromosome 10q11.2 associated with prostate cancer susceptibility. *Proc Natl Acad Sci U S A* 106**,** 7933-7938.

Maller, J.B., Fagerness, J.A., Reynolds, R.C., Neale, B.M., Daly, M.J., and Seddon, J.M. (2007). Variation in complement factor 3 is associated with risk of age-related macular degeneration. *Nat Genet* 39**,** 1200-1201.

Meigs, J.B., Shrader, P., Sullivan, L.M., Mcateer, J.B., Fox, C.S., Dupuis, J., Manning, A.K., Florez, J.C., Wilson, P.W., D'agostino, R.B., Sr., and Cupples, L.A. (2008). Genotype score in addition to common risk factors for prediction of type 2 diabetes. *N Engl J Med* 359**,** 2208-2219. doi: 10.1056/NEJMoa0804742.

Miyake, K., Horikawa, Y., Hara, K., Yasuda, K., Osawa, H., Furuta, H., Hirota, Y., Yamagata, K., Hinokio, Y., Oka, Y., Iwasaki, N., Iwamoto, Y., Yamada, Y., Seino, Y., Maegawa, H., Kashiwagi, A., Yamamoto, K., Tokunaga, K., Takeda, J., Makino, H., Nanjo, K., Kadowaki, T., and Kasuga, M. (2008). Association of TCF7L2 polymorphisms with susceptibility to type 2 diabetes in 4,087 Japanese subjects. *J Hum Genet* 53**,** 174-180. doi: 10.1007/s10038-007-0231-5 [doi].

Neale, B.M., Fagerness, J., Reynolds, R., Sobrin, L., Parker, M., Raychaudhuri, S., Tan, P.L., Oh, E.C., Merriam, J.E., Souied, E., Bernstein, P.S., Li, B., Frederick, J.M., Zhang, K., Brantley, M.A., Jr., Lee, A.Y., Zack, D.J., Campochiaro, B., Campochiaro, P., Ripke, S., Smith, R.T., Barile, G.R., Katsanis, N., Allikmets, R., Daly, M.J., and Seddon, J.M. (2010). Genome-wide association study of advanced age-related macular degeneration identifies a role of the hepatic lipase gene (LIPC). *Proc Natl Acad Sci U S A* 107**,** 7395-7400.

Ng, M.C., Park, K.S., Oh, B., Tam, C.H., Cho, Y.M., Shin, H.D., Lam, V.K., Ma, R.C., So, W.Y., Cho, Y.S., Kim, H.L., Lee, H.K., Chan, J.C., and Cho, N.H. (2008). Implication of genetic variants near TCF7L2, SLC30A8, HHEX, CDKAL1, CDKN2A/B, IGF2BP2, and FTO in type 2 diabetes and obesity in 6,719 Asians. *Diabetes* 57**,** 2226-2233. doi: db07-1583 [pii]

10.2337/db07-1583 [doi].

Parkes, M., Barrett, J.C., Prescott, N.J., Tremelling, M., Anderson, C.A., Fisher, S.A., Roberts, R.G., Nimmo, E.R., Cummings, F.R., Soars, D., Drummond, H., Lees, C.W., Khawaja, S.A., Bagnall, R., Burke, D.A., Todhunter, C.E., Ahmad, T., Onnie, C.M., Mcardle, W., Strachan, D., Bethel, G., Bryan, C., Lewis, C.M., Deloukas, P., Forbes, A., Sanderson, J., Jewell, D.P., Satsangi, J., Mansfield, J.C., Wellcome Trust Case Control, C., Cardon, L., and Mathew, C.G. (2007). Sequence variants in the autophagy gene IRGM and multiple other replicating loci contribute to Crohn's disease susceptibility. *Nat Genet* 39**,** 830-832.

Prokopenko, I., Langenberg, C., Florez, J.C., Saxena, R., Soranzo, N., Thorleifsson, G., Loos, R.J., Manning, A.K., Jackson, A.U., Aulchenko, Y., Potter, S.C., Erdos, M.R., Sanna, S., Hottenga, J.J., Wheeler, E., Kaakinen, M., Lyssenko, V., Chen, W.M., Ahmadi, K., Beckmann, J.S., Bergman, R.N., Bochud, M., Bonnycastle, L.L., Buchanan, T.A., Cao, A., Cervino, A., Coin, L., Collins, F.S., Crisponi, L., De Geus, E.J., Dehghan, A., Deloukas, P., Doney, A.S., Elliott, P., Freimer, N., Gateva, V., Herder, C., Hofman, A., Hughes, T.E., Hunt, S., Illig, T., Inouye, M., Isomaa, B., Johnson, T., Kong, A., Krestyaninova, M., Kuusisto, J., Laakso, M., Lim, N., Lindblad, U., Lindgren, C.M., Mccann, O.T., Mohlke, K.L., Morris, A.D., Naitza, S., Orru, M., Palmer, C.N., Pouta, A., Randall, J., Rathmann, W., Saramies, J., Scheet, P., Scott, L.J., Scuteri, A., Sharp, S., Sijbrands, E., Smit, J.H., Song, K., Steinthorsdottir, V., Stringham, H.M., Tuomi, T., Tuomilehto, J., Uitterlinden, A.G., Voight, B.F., Waterworth, D., Wichmann, H.E., Willemsen, G., Witteman, J.C., Yuan, X., Zhao, J.H., Zeggini, E., Schlessinger, D., Sandhu, M., Boomsma, D.I., Uda, M., Spector, T.D., Penninx, B.W., Altshuler, D., Vollenweider, P., Jarvelin, M.R., Lakatta, E., Waeber, G., Fox, C.S., Peltonen, L., Groop, L.C., Mooser, V., Cupples, L.A., Thorsteinsdottir, U., Boehnke, M., Barroso, I., et al. (2009). Variants in MTNR1B influence fasting glucose levels. *Nat Genet* 41**,** 77-81. doi: ng.290 [pii]

10.1038/ng.290 [doi].

Rivera, A., Fisher, S.A., Fritsche, L.G., Keilhauer, C.N., Lichtner, P., Meitinger, T., and Weber, B.H. (2005). Hypothetical LOC387715 is a second major susceptibility gene for age-related macular degeneration, contributing independently of complement factor H to disease risk. *Hum Mol Genet* 14**,** 3227-3236.

Sandhu, M.S., Weedon, M.N., Fawcett, K.A., Wasson, J., Debenham, S.L., Daly, A., Lango, H., Frayling, T.M., Neumann, R.J., Sherva, R., Blech, I., Pharoah, P.D., Palmer, C.N., Kimber, C., Tavendale, R., Morris, A.D., Mccarthy, M.I., Walker, M., Hitman, G., Glaser, B., Permutt, M.A., Hattersley, A.T., Wareham, N.J., and Barroso, I. (2007). Common variants in WFS1 confer risk of type 2 diabetes. *Nat Genet* 39**,** 951-953. doi: ng2067 [pii]

10.1038/ng2067 [doi].

Saxena, R., Voight, B.F., Lyssenko, V., Burtt, N.P., De Bakker, P.I., Chen, H., Roix, J.J., Kathiresan, S., Hirschhorn, J.N., Daly, M.J., Hughes, T.E., Groop, L., Altshuler, D., Almgren, P., Florez, J.C., Meyer, J., Ardlie, K., Bengtsson Bostrom, K., Isomaa, B., Lettre, G., Lindblad, U., Lyon, H.N., Melander, O., Newton-Cheh, C., Nilsson, P., Orho-Melander, M., Rastam, L., Speliotes, E.K., Taskinen, M.R., Tuomi, T., Guiducci, C., Berglund, A., Carlson, J., Gianniny, L., Hackett, R., Hall, L., Holmkvist, J., Laurila, E., Sjogren, M., Sterner, M., Surti, A., Svensson, M., Tewhey, R., Blumenstiel, B., Parkin, M., Defelice, M., Barry, R., Brodeur, W., Camarata, J., Chia, N., Fava, M., Gibbons, J., Handsaker, B., Healy, C., Nguyen, K., Gates, C., Sougnez, C., Gage, D., Nizzari, M., Gabriel, S.B., Chirn, G.W., Ma, Q., Parikh, H., Richardson, D., Ricke, D., and Purcell, S. (2007). Genome-wide association analysis identifies loci for type 2 diabetes and triglyceride levels. *Science* 316**,** 1331-1336. doi: 1142358 [pii]

10.1126/science.1142358 [doi].

Scott, L.J., Mohlke, K.L., Bonnycastle, L.L., Willer, C.J., Li, Y., Duren, W.L., Erdos, M.R., Stringham, H.M., Chines, P.S., Jackson, A.U., Prokunina-Olsson, L., Ding, C.J., Swift, A.J., Narisu, N., Hu, T., Pruim, R., Xiao, R., Li, X.Y., Conneely, K.N., Riebow, N.L., Sprau, A.G., Tong, M., White, P.P., Hetrick, K.N., Barnhart, M.W., Bark, C.W., Goldstein, J.L., Watkins, L., Xiang, F., Saramies, J., Buchanan, T.A., Watanabe, R.M., Valle, T.T., Kinnunen, L., Abecasis, G.R., Pugh, E.W., Doheny, K.F., Bergman, R.N., Tuomilehto, J., Collins, F.S., and Boehnke, M. (2007). A genome-wide association study of type 2 diabetes in Finns detects multiple susceptibility variants. *Science* 316**,** 1341-1345.

Shaat, N., Karlsson, E., Lernmark, A., Ivarsson, S., Lynch, K., Parikh, H., Almgren, P., Berntorp, K., and Groop, L. (2006). Common variants in MODY genes increase the risk of gestational diabetes mellitus. *Diabetologia* 49**,** 1545-1551.

Sladek, R., Rocheleau, G., Rung, J., Dina, C., Shen, L., Serre, D., Boutin, P., Vincent, D., Belisle, A., Hadjadj, S., Balkau, B., Heude, B., Charpentier, G., Hudson, T.J., Montpetit, A., Pshezhetsky, A.V., Prentki, M., Posner, B.I., Balding, D.J., Meyre, D., Polychronakos, C., and Froguel, P. (2007). A genome-wide association study identifies novel risk loci for type 2 diabetes. *Nature* 445**,** 881-885.

Song, Y., Niu, T., Manson, J.E., Kwiatkowski, D.J., and Liu, S. (2004). Are variants in the CAPN10 gene related to risk of type 2 diabetes? A quantitative assessment of population and family-based association studies. *Am J Hum Genet* 74**,** 208-222.

Sparso, T., Andersen, G., Nielsen, T., Burgdorf, K.S., Gjesing, A.P., Nielsen, A.L., Albrechtsen, A., Rasmussen, S.S., Jorgensen, T., Borch-Johnsen, K., Sandbaek, A., Lauritzen, T., Madsbad, S., Hansen, T., and Pedersen, O. (2008). The GCKR rs780094 polymorphism is associated with elevated fasting serum triacylglycerol, reduced fasting and OGTT-related insulinaemia, and reduced risk of type 2 diabetes. *Diabetologia* 51**,** 70-75.

Steinthorsdottir, V., Thorleifsson, G., Reynisdottir, I., Benediktsson, R., Jonsdottir, T., Walters, G.B., Styrkarsdottir, U., Gretarsdottir, S., Emilsson, V., Ghosh, S., Baker, A., Snorradottir, S., Bjarnason, H., Ng, M.C., Hansen, T., Bagger, Y., Wilensky, R.L., Reilly, M.P., Adeyemo, A., Chen, Y., Zhou, J., Gudnason, V., Chen, G., Huang, H., Lashley, K., Doumatey, A., So, W.Y., Ma, R.C., Andersen, G., Borch-Johnsen, K., Jorgensen, T., Van Vliet-Ostaptchouk, J.V., Hofker, M.H., Wijmenga, C., Christiansen, C., Rader, D.J., Rotimi, C., Gurney, M., Chan, J.C., Pedersen, O., Sigurdsson, G., Gulcher, J.R., Thorsteinsdottir, U., Kong, A., and Stefansson, K. (2007). A variant in CDKAL1 influences insulin response and risk of type 2 diabetes. *Nat Genet* 39**,** 770-775.

Sun, J., Zheng, S.L., Wiklund, F., Isaacs, S.D., Li, G., Wiley, K.E., Kim, S.T., Zhu, Y., Zhang, Z., Hsu, F.C., Turner, A.R., Stattin, P., Liu, W., Kim, J.W., Duggan, D., Carpten, J., Isaacs, W., Gronberg, H., Xu, J., and Chang, B.L. (2009). Sequence variants at 22q13 are associated with prostate cancer risk. *Cancer Res* 69**,** 10-15.

Sun, J., Zheng, S.L., Wiklund, F., Isaacs, S.D., Purcell, L.D., Gao, Z., Hsu, F.C., Kim, S.T., Liu, W., Zhu, Y., Stattin, P., Adami, H.O., Wiley, K.E., Dimitrov, L., Sun, J., Li, T., Turner, A.R., Adams, T.S., Adolfsson, J., Johansson, J.E., Lowey, J., Trock, B.J., Partin, A.W., Walsh, P.C., Trent, J.M., Duggan, D., Carpten, J., Chang, B.L., Gronberg, H., Isaacs, W.B., and Xu, J. (2008). Evidence for two independent prostate cancer risk-associated loci in the HNF1B gene at 17q12. *Nat Genet* 40**,** 1153-1155.

Tenesa, A., Farrington, S.M., Prendergast, J.G., Porteous, M.E., Walker, M., Haq, N., Barnetson, R.A., Theodoratou, E., Cetnarskyj, R., Cartwright, N., Semple, C., Clark, A.J., Reid, F.J., Smith, L.A., Kavoussanakis, K., Koessler, T., Pharoah, P.D., Buch, S., Schafmayer, C., Tepel, J., Schreiber, S., Volzke, H., Schmidt, C.O., Hampe, J., Chang-Claude, J., Hoffmeister, M., Brenner, H., Wilkening, S., Canzian, F., Capella, G., Moreno, V., Deary, I.J., Starr, J.M., Tomlinson, I.P., Kemp, Z., Howarth, K., Carvajal-Carmona, L., Webb, E., Broderick, P., Vijayakrishnan, J., Houlston, R.S., Rennert, G., Ballinger, D., Rozek, L., Gruber, S.B., Matsuda, K., Kidokoro, T., Nakamura, Y., Zanke, B.W., Greenwood, C.M., Rangrej, J., Kustra, R., Montpetit, A., Hudson, T.J., Gallinger, S., Campbell, H., and Dunlop, M.G. (2008). Genome-wide association scan identifies a colorectal cancer susceptibility locus on 11q23 and replicates risk loci at 8q24 and 18q21. *Nat Genet* 40**,** 631-637.

Thomas, G., Jacobs, K.B., Yeager, M., Kraft, P., Wacholder, S., Orr, N., Yu, K., Chatterjee, N., Welch, R., Hutchinson, A., Crenshaw, A., Cancel-Tassin, G., Staats, B.J., Wang, Z., Gonzalez-Bosquet, J., Fang, J., Deng, X., Berndt, S.I., Calle, E.E., Feigelson, H.S., Thun, M.J., Rodriguez, C., Albanes, D., Virtamo, J., Weinstein, S., Schumacher, F.R., Giovannucci, E., Willett, W.C., Cussenot, O., Valeri, A., Andriole, G.L., Crawford, E.D., Tucker, M., Gerhard, D.S., Fraumeni, J.F., Jr., Hoover, R., Hayes, R.B., Hunter, D.J., and Chanock, S.J. (2008). Multiple loci identified in a genome-wide association study of prostate cancer. *Nat Genet* 40**,** 310-315.

Todd, J.A., Walker, N.M., Cooper, J.D., Smyth, D.J., Downes, K., Plagnol, V., Bailey, R., Nejentsev, S., Field, S.F., Payne, F., Lowe, C.E., Szeszko, J.S., Hafler, J.P., Zeitels, L., Yang, J.H., Vella, A., Nutland, S., Stevens, H.E., Schuilenburg, H., Coleman, G., Maisuria, M., Meadows, W., Smink, L.J., Healy, B., Burren, O.S., Lam, A.A., Ovington, N.R., Allen, J., Adlem, E., Leung, H.T., Wallace, C., Howson, J.M., Guja, C., Ionescu-Tirgoviste, C., Genetics of Type 1 Diabetes In, F., Simmonds, M.J., Heward, J.M., Gough, S.C., Wellcome Trust Case Control, C., Dunger, D.B., Wicker, L.S., and Clayton, D.G. (2007). Robust associations of four new chromosome regions from genome-wide analyses of type 1 diabetes. *Nat Genet* 39**,** 857-864.

Tomlinson, I., Webb, E., Carvajal-Carmona, L., Broderick, P., Kemp, Z., Spain, S., Penegar, S., Chandler, I., Gorman, M., Wood, W., Barclay, E., Lubbe, S., Martin, L., Sellick, G., Jaeger, E., Hubner, R., Wild, R., Rowan, A., Fielding, S., Howarth, K., Consortium, C., Silver, A., Atkin, W., Muir, K., Logan, R., Kerr, D., Johnstone, E., Sieber, O., Gray, R., Thomas, H., Peto, J., Cazier, J.B., and Houlston, R. (2007). A genome-wide association scan of tag SNPs identifies a susceptibility variant for colorectal cancer at 8q24.21. *Nat Genet* 39**,** 984-988.

Tomlinson, I.P., Webb, E., Carvajal-Carmona, L., Broderick, P., Howarth, K., Pittman, A.M., Spain, S., Lubbe, S., Walther, A., Sullivan, K., Jaeger, E., Fielding, S., Rowan, A., Vijayakrishnan, J., Domingo, E., Chandler, I., Kemp, Z., Qureshi, M., Farrington, S.M., Tenesa, A., Prendergast, J.G., Barnetson, R.A., Penegar, S., Barclay, E., Wood, W., Martin, L., Gorman, M., Thomas, H., Peto, J., Bishop, D.T., Gray, R., Maher, E.R., Lucassen, A., Kerr, D., Evans, D.G., Consortium, C., Schafmayer, C., Buch, S., Volzke, H., Hampe, J., Schreiber, S., John, U., Koessler, T., Pharoah, P., Van Wezel, T., Morreau, H., Wijnen, J.T., Hopper, J.L., Southey, M.C., Giles, G.G., Severi, G., Castellvi-Bel, S., Ruiz-Ponte, C., Carracedo, A., Castells, A., Consortium, E., Forsti, A., Hemminki, K., Vodicka, P., Naccarati, A., Lipton, L., Ho, J.W., Cheng, K.K., Sham, P.C., Luk, J., Agundez, J.A., Ladero, J.M., De La Hoya, M., Caldes, T., Niittymaki, I., Tuupanen, S., Karhu, A., Aaltonen, L., Cazier, J.B., Campbell, H., Dunlop, M.G., and Houlston, R.S. (2008). A genome-wide association study identifies colorectal cancer susceptibility loci on chromosomes 10p14 and 8q23.3. *Nat Genet* 40**,** 623-630.

Unoki, H., Takahashi, A., Kawaguchi, T., Hara, K., Horikoshi, M., Andersen, G., Ng, D.P., Holmkvist, J., Borch-Johnsen, K., Jorgensen, T., Sandbaek, A., Lauritzen, T., Hansen, T., Nurbaya, S., Tsunoda, T., Kubo, M., Babazono, T., Hirose, H., Hayashi, M., Iwamoto, Y., Kashiwagi, A., Kaku, K., Kawamori, R., Tai, E.S., Pedersen, O., Kamatani, N., Kadowaki, T., Kikkawa, R., Nakamura, Y., and Maeda, S. (2008). SNPs in KCNQ1 are associated with susceptibility to type 2 diabetes in East Asian and European populations. *Nat Genet* 40**,** 1098-1102.

Van Hoek, M., Dehghan, A., Witteman, J.C., Van Duijn, C.M., Uitterlinden, A.G., Oostra, B.A., Hofman, A., Sijbrands, E.J., and Janssens, A.C. (2008). Predicting type 2 diabetes based on polymorphisms from genome-wide association studies: a population-based study. *Diabetes* 57**,** 3122-3128. doi: 10.2337/db08-0425.

Voight, B.F., Scott, L.J., Steinthorsdottir, V., Morris, A.P., Dina, C., Welch, R.P., Zeggini, E., Huth, C., Aulchenko, Y.S., Thorleifsson, G., Mcculloch, L.J., Ferreira, T., Grallert, H., Amin, N., Wu, G., Willer, C.J., Raychaudhuri, S., Mccarroll, S.A., Langenberg, C., Hofmann, O.M., Dupuis, J., Qi, L., Segre, A.V., Van Hoek, M., Navarro, P., Ardlie, K., Balkau, B., Benediktsson, R., Bennett, A.J., Blagieva, R., Boerwinkle, E., Bonnycastle, L.L., Bengtsson Bostrom, K., Bravenboer, B., Bumpstead, S., Burtt, N.P., Charpentier, G., Chines, P.S., Cornelis, M., Couper, D.J., Crawford, G., Doney, A.S., Elliott, K.S., Elliott, A.L., Erdos, M.R., Fox, C.S., Franklin, C.S., Ganser, M., Gieger, C., Grarup, N., Green, T., Griffin, S., Groves, C.J., Guiducci, C., Hadjadj, S., Hassanali, N., Herder, C., Isomaa, B., Jackson, A.U., Johnson, P.R., Jorgensen, T., Kao, W.H., Klopp, N., Kong, A., Kraft, P., Kuusisto, J., Lauritzen, T., Li, M., Lieverse, A., Lindgren, C.M., Lyssenko, V., Marre, M., Meitinger, T., Midthjell, K., Morken, M.A., Narisu, N., Nilsson, P., Owen, K.R., Payne, F., Perry, J.R., Petersen, A.K., Platou, C., Proenca, C., Prokopenko, I., Rathmann, W., Rayner, N.W., Robertson, N.R., Rocheleau, G., Roden, M., Sampson, M.J., Saxena, R., Shields, B.M., Shrader, P., Sigurdsson, G., Sparso, T., Strassburger, K., Stringham, H.M., Sun, Q., Swift, A.J., Thorand, B., et al. (2010). Twelve type 2 diabetes susceptibility loci identified through large-scale association analysis. *Nat Genet* 42**,** 579-589.

Wang, C., Hu, C., Zhang, R., Bao, Y., Ma, X., Lu, J., Qin, W., Shao, X., Lu, J., Xu, J., Lu, H., Xiang, K., and Jia, W. (2009). Common variants of hepatocyte nuclear factor 1beta are associated with type 2 diabetes in a Chinese population. *Diabetes* 58**,** 1023-1027.

Wasson, J., and Permutt, M.A. (2008). Candidate gene studies reveal that the WFS1 gene joins the expanding list of novel type 2 diabetes genes. *Diabetologia* 51**,** 391-393.

Weedon, M.N., Owen, K.R., Shields, B., Hitman, G., Walker, M., Mccarthy, M.I., Hattersley, A.T., and Frayling, T.M. (2005). A large-scale association analysis of common variation of the HNF1alpha gene with type 2 diabetes in the U.K. Caucasian population. *Diabetes* 54**,** 2487-2491.

Winckler, W., Weedon, M.N., Graham, R.R., Mccarroll, S.A., Purcell, S., Almgren, P., Tuomi, T., Gaudet, D., Bostrom, K.B., Walker, M., Hitman, G., Hattersley, A.T., Mccarthy, M.I., Ardlie, K.G., Hirschhorn, J.N., Daly, M.J., Frayling, T.M., Groop, L., and Altshuler, D. (2007). Evaluation of common variants in the six known maturity-onset diabetes of the young (MODY) genes for association with type 2 diabetes. *Diabetes* 56**,** 685-693.

Yasuda, K., Miyake, K., Horikawa, Y., Hara, K., Osawa, H., Furuta, H., Hirota, Y., Mori, H., Jonsson, A., Sato, Y., Yamagata, K., Hinokio, Y., Wang, H.Y., Tanahashi, T., Nakamura, N., Oka, Y., Iwasaki, N., Iwamoto, Y., Yamada, Y., Seino, Y., Maegawa, H., Kashiwagi, A., Takeda, J., Maeda, E., Shin, H.D., Cho, Y.M., Park, K.S., Lee, H.K., Ng, M.C., Ma, R.C., So, W.Y., Chan, J.C., Lyssenko, V., Tuomi, T., Nilsson, P., Groop, L., Kamatani, N., Sekine, A., Nakamura, Y., Yamamoto, K., Yoshida, T., Tokunaga, K., Itakura, M., Makino, H., Nanjo, K., Kadowaki, T., and Kasuga, M. (2008). Variants in KCNQ1 are associated with susceptibility to type 2 diabetes mellitus. *Nat Genet* 40**,** 1092-1097.

Yeager, M., Orr, N., Hayes, R.B., Jacobs, K.B., Kraft, P., Wacholder, S., Minichiello, M.J., Fearnhead, P., Yu, K., Chatterjee, N., Wang, Z., Welch, R., Staats, B.J., Calle, E.E., Feigelson, H.S., Thun, M.J., Rodriguez, C., Albanes, D., Virtamo, J., Weinstein, S., Schumacher, F.R., Giovannucci, E., Willett, W.C., Cancel-Tassin, G., Cussenot, O., Valeri, A., Andriole, G.L., Gelmann, E.P., Tucker, M., Gerhard, D.S., Fraumeni, J.F., Jr., Hoover, R., Hunter, D.J., Chanock, S.J., and Thomas, G. (2007). Genome-wide association study of prostate cancer identifies a second risk locus at 8q24. *Nat Genet* 39**,** 645-649.

Zeggini, E., Scott, L.J., Saxena, R., Voight, B.F., Marchini, J.L., Hu, T., De Bakker, P.I., Abecasis, G.R., Almgren, P., Andersen, G., Ardlie, K., Bostrom, K.B., Bergman, R.N., Bonnycastle, L.L., Borch-Johnsen, K., Burtt, N.P., Chen, H., Chines, P.S., Daly, M.J., Deodhar, P., Ding, C.J., Doney, A.S., Duren, W.L., Elliott, K.S., Erdos, M.R., Frayling, T.M., Freathy, R.M., Gianniny, L., Grallert, H., Grarup, N., Groves, C.J., Guiducci, C., Hansen, T., Herder, C., Hitman, G.A., Hughes, T.E., Isomaa, B., Jackson, A.U., Jorgensen, T., Kong, A., Kubalanza, K., Kuruvilla, F.G., Kuusisto, J., Langenberg, C., Lango, H., Lauritzen, T., Li, Y., Lindgren, C.M., Lyssenko, V., Marvelle, A.F., Meisinger, C., Midthjell, K., Mohlke, K.L., Morken, M.A., Morris, A.D., Narisu, N., Nilsson, P., Owen, K.R., Palmer, C.N., Payne, F., Perry, J.R., Pettersen, E., Platou, C., Prokopenko, I., Qi, L., Qin, L., Rayner, N.W., Rees, M., Roix, J.J., Sandbaek, A., Shields, B., Sjogren, M., Steinthorsdottir, V., Stringham, H.M., Swift, A.J., Thorleifsson, G., Thorsteinsdottir, U., Timpson, N.J., Tuomi, T., Tuomilehto, J., Walker, M., Watanabe, R.M., Weedon, M.N., Willer, C.J., Wellcome Trust Case Control, C., Illig, T., Hveem, K., Hu, F.B., Laakso, M., Stefansson, K., Pedersen, O., Wareham, N.J., Barroso, I., Hattersley, A.T., Collins, F.S., Groop, L., Mccarthy, M.I., Boehnke, M., and Altshuler, D. (2008). Meta-analysis of genome-wide association data and large-scale replication identifies additional susceptibility loci for type 2 diabetes. *Nat Genet* 40**,** 638-645.

Zeggini, E., Weedon, M.N., Lindgren, C.M., Frayling, T.M., Elliott, K.S., Lango, H., Timpson, N.J., Perry, J.R., Rayner, N.W., Freathy, R.M., Barrett, J.C., Shields, B., Morris, A.P., Ellard, S., Groves, C.J., Harries, L.W., Marchini, J.L., Owen, K.R., Knight, B., Cardon, L.R., Walker, M., Hitman, G.A., Morris, A.D., Doney, A.S., Wellcome Trust Case Control, C., Mccarthy, M.I., and Hattersley, A.T. (2007). Replication of genome-wide association signals in UK samples reveals risk loci for type 2 diabetes. *Science* 316**,** 1336-1341.

Zheng, S.L., Stevens, V.L., Wiklund, F., Isaacs, S.D., Sun, J., Smith, S., Pruett, K., Wiley, K.E., Kim, S.T., Zhu, Y., Zhang, Z., Hsu, F.C., Turner, A.R., Johansson, J.E., Liu, W., Kim, J.W., Chang, B.L., Duggan, D., Carpten, J., Rodriguez, C., Isaacs, W., Gronberg, H., and Xu, J. (2009). Two independent prostate cancer risk-associated Loci at 11q13. *Cancer Epidemiol Biomarkers Prev* 18**,** 1815-1820.
